# New Crocodyliforms from Southwestern Europe and Definition of a Diverse Clade of European Late Cretaceous Basal Eusuchians

**DOI:** 10.1371/journal.pone.0140679

**Published:** 2015-11-04

**Authors:** Iván Narváez, Christopher A. Brochu, Fernando Escaso, Adán Pérez-García, Francisco Ortega

**Affiliations:** 1 Grupo de Biología Evolutiva, Universidad Nacional de Educación a Distancia, Madrid, Madrid, Spain; 2 Department of Earth and Environmental Sciences, University of Iowa, Iowa City, Iowa, United States of America; Royal Belgian Institute of Natural Sciences, BELGIUM

## Abstract

The late Campanian-early Maastrichtian site of Lo Hueco (Cuenca, Spain) has provided a set of well-preserved crocodyliform skull and lower jaw remains, which are described here and assigned to a new basal eusuchian taxon, *Lohuecosuchus megadontos* gen. et sp. nov. The reevaluation of a complete skull from the synchronous site of Fox-Amphoux (Department of Var, France) allows us to define a second species of this new genus. Phylogenetic analysis places *Lohuecosuchus* in a clade exclusively composed by European Late Cretaceous taxa. This new clade, defined here as Allodaposuchidae, is recognized as the sister group of Hylaeochampsidae, also comprised of European Cretaceous forms. Allodaposuchidae and Hylaeochampsidae are grouped in a clade identified as the sister group of Crocodylia, the only crocodyliform lineage that reaches our days. Allodaposuchidae shows a vicariant distribution pattern in the European Late Cretaceous archipelago, with several Ibero-Armorican forms more closely related to each other than with to Romanian *Allodaposuchus precedens*.

## Introduction

The emergence of Eusuchia, the group that gave rise to Crocodylia, involved major changes in the skeletal design of neosuchian crocodyliforms during the Early Cretaceous. The oldest unambiguous eusuchian, *Hylaeochampsa vectiana* [1], is from the Early Cretaceous (Barremian) of the Isle of Wight (England), but most known eusuchians outside Crocodylia are from the Late Cretaceous of Europe [2–8]. Their remains are relatively scarce and, until recently, were poorly known. Due to this, the phylogenetic relationships among several of them, and of these forms with Crocodylia, are unclear [9–12].

Several European Late Cretaceous forms were preliminarily attributed to members of Crocodylia. These putative crocodylians included two taxa, *Musturzabalsuchus buffetauti* [13] from Spain and *Massaliasuchus affuvelensis* [14] from France, referred to Alligatoroidea in some analyses [13, 15, 16]; the European representatives of the putative gavialoid *Thoracosaurus* [17, 18], from France, Netherlands and Crimea; and *Arenysuchus gascabadiolorum* [4] from Spain, initially considered a basal crocodyloid [4]. In addition, some authors proposed that the members of *Acynodon* [13], a taxon from the Campanian-Maastrichtian of Spain, France and Italy, belong to Alligatoroidea [13, 15, 19, 20]; and that the species assigned to *Allodaposuchus* [21] could also belong to that clade [22], or be members of Crocodylia [6].

The taxonomic status of most of these crocodyliforms has recently been considered as problematic [3, 23, 24]. The hypothesis currently supported by most authors groups *Acynodon* [13], *Hylaeochampsa* [1] and *Iharkutosuchus* [25] from the Santonian of Hungary in Hylaeochampsidae, a clade of non-crocodylian eusuchians [5, 12, 26, 27]. In fact, the type species of *Allodaposuchus* (Romanian *Allodaposuchus precedens* [21]), is generally considered to be closely related to the hylaeochampsids [5, 9, 11, 12, 26, 28]. New discoveries provide some European Late Cretaceous specimens that could be related to *Al*. *precedens*: a skull from the French locality of Fox-Amphoux, referred by Martin (2010) [22] to *Allodaposuchus* cf. *Al*. *precedens*; a series of cranial remains and a few postcranial elements from Velaux-La Bastide Neuve, in Southern France, assigned to an ontogenetic series of *Al*. *precedens* [8]; and the types and only known specimens of the Iberian *Arenysuchus gascabadiolorum* [4], *Allodaposuchus subjuniperus* [5], *Allodaposuchus hulki* [7] and *Allodaposuchus palustris* [6], whose validity has been questioned [8]. In addition, two problematic taxa have recently been considered as related to *Al*. *precedens*: *Massaliasuchus affuvelensis* and *Musturzabalsuchus buffetauti* [24, 29, 30].

Information about these forms, all represented by a single individual except the ontogenetic series from Velaux-La Bastide Neuve [8], is based on material varying in completeness from highly fragmentary remains (*Ma*. *affuvelensis*, *Mu*. *buffetauti*, *Al*. *palustris*, *Al*. *hulki*), to partial (*Ar*. *gascabadiolorum*) or almost complete skulls (*Al*. *subjuniperus* and the specimen from Fox-Amphoux). The only cranial material from the type locality of *Allodaposuchus precedens*, Vălioara (Romania), corresponds to the posterodorsal region of a skull [21], though Delfino et al. [28] attributed a complete skull, from Oarda de Jos (Romania) to this species. Therefore, the information available on most of these taxa is limited. In addition, most of the specimens referred to *Allodaposuchus* have been identified as adult individuals, except some specimens from Velaux-La Bastide Neuve and probably the holotype and only known specimen of *Ar*. *gascabadiolorum* [8, 30]. The discovery of additional material, analyzed in a comprehensive phylogenetic context, will be critical for understanding ancestral morphological conditions for Crocodylia [3, 24].

**Fig 1:**
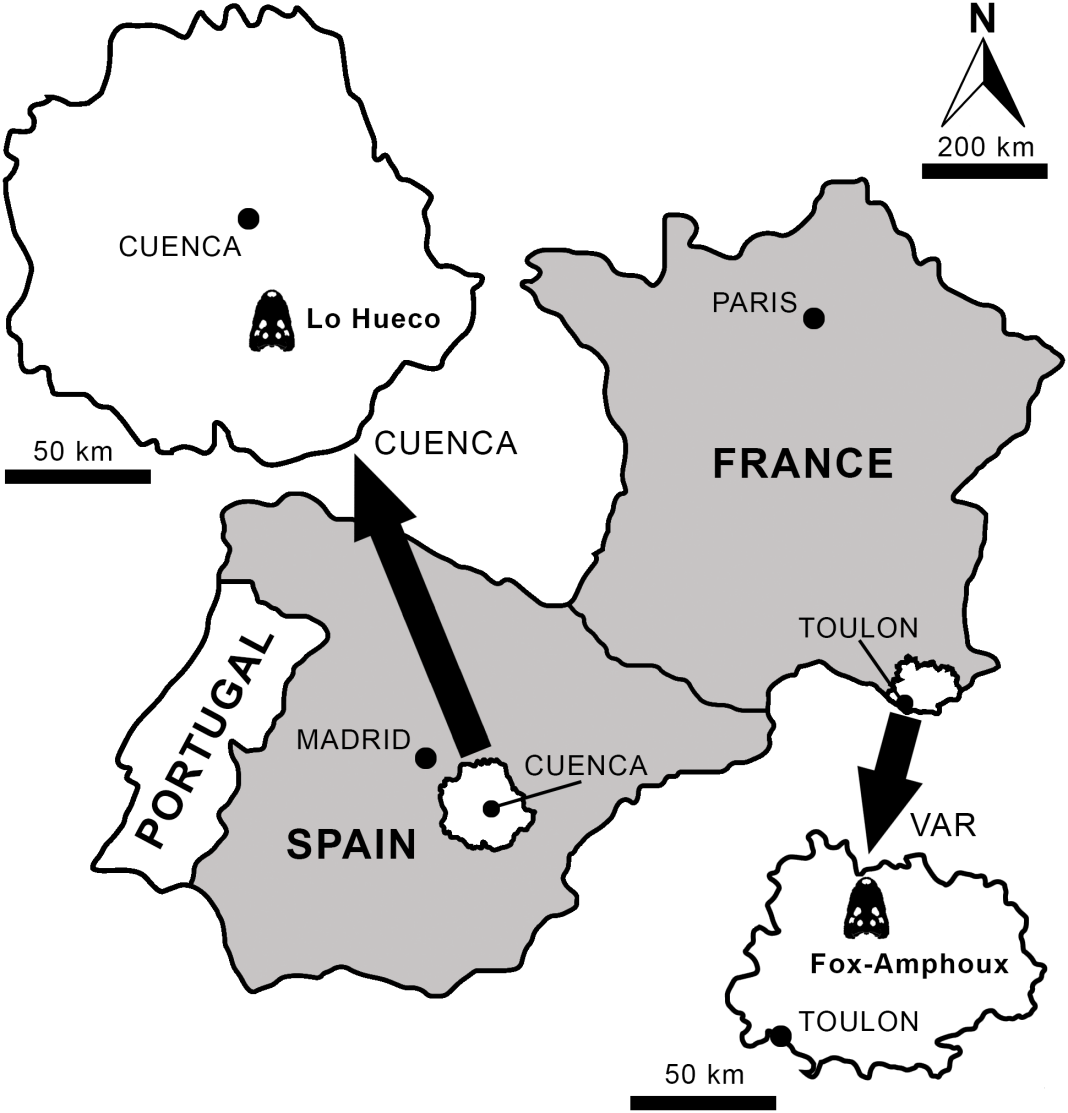
Geographic location of the sites where the European crocodyliform *Lohuecosuchus* gen. nov. has been identified. Lo Hueco (Cuenca, Spain), type locality of *Lohuecosuchus megadontos* sp. nov.; and Fox-Amphoux (Var, France), type locality of *Lohuecosuchus mechinorum* sp. nov.

The Lo Hueco fossil site (Fig 1), discovered in 2007 at the village of Fuentes (Cuenca, Spain), has yielded a rich fossil assemblage consisting of plant, invertebrate and, especially, vertebrate remains [31–33]. The vertebrates are represented by teleostean fishes, amphibians, panpleurodiran (bothremydid) and pancryptodiran turtles, squamates (including pythonomorphs), eusuchian crocodyliforms, rhabdodontid ornithopods, theropods (mainly dromaeosaurids), and titanosaurid sauropods [31–40]. Crocodyliforms are relatively abundant at the locality. The collection includes several skulls and lower jaws from non-Crocodylia eusuchians with features suggesting a close phylogenetic relationship with the genus *Allodaposuchus* [35]. Several of these elements can be recognized as belonging to a medium sized crocodyliform with a relatively wide and rounded rostrum. This form is described here, *Lohuecosuchus megadontos* gen. et sp. nov.

The material from Lo Hueco presented here provides a wealth of information based on several well-preserved specimens belonging to a single taxon. This contrasts with the limited information provided by most other taxa related to *Al*. *precedens*. Therefore, a detailed description of the skull of the new taxon is provided. In addition, several complete lower jaws are described, the holotype being composed by a complete skull and its right lower jaw. This is remarkable, because among the taxa related to *Al*. *precedens*, mandibular material (usually fragmentary) has only been recognized in *Mu*. *buffetauti*, *Ma*. *affuvelensis*, *Al*. *palustris*, *Al*. *hulki* and specimens from Velaux-La Bastide Neuve. The abundant material from the Lo Hueco form allows further assessment of variability in a member of this lineage based on specimens from the same locality.

This study not only allows us to recognize a new Iberian taxon, but to establish a new French species closely related to it, *Lohuecosuchus mechinorum* sp. nov. The inclusion of these taxa in a phylogenetic analysis allows us to establish a new clade, Allodaposuchidae, including several European Late Cretaceous crocodyliforms. The controversial systematic position of these forms in relation to other eusuchians, including Hylaeochampsidae and Crocodylia, is evaluated.

### Nomenclature Acts

The electronic edition of this article conforms to the requirements of the amended International Code of Zoological Nomenclature, and hence the new names contained herein are available under that Code from the electronic edition of this article. This published work and the nomenclatural acts it contains have been registered in ZooBank, the online registration system for the ICZN. The ZooBank LSIDs (Life Science Identifiers) can be resolved and the associated information viewed through any standard web browser by appending the LSID to the prefix “http://zoobank.org/”. The LSID for this publication is: urn:lsid:zoobank.org:pub:2D30C597-B785-40FD-B199-4D4309ABCF39. The electronic edition of this work was published in a journal with an ISSN, and has been archived and is available from the following digital repositories: PubMed Central and LOCKSS.

### Paleontological Ethics Statements

The type series (holotype HUE-04498, and paratypes HUE-02920, HUE-04263, HUE-04378 and HUE-05161) of *Lohuecosuchus megadontos* gen et sp nov. described in this paper is deposited in the collections of the Museo de las Ciencias de Castilla-La Mancha (Cuenca, Spain). All necessary permits were obtained for the described study, which complied with all relevant regulations. The type series specimens were collected under permits obtained from the Dirección General de Patrimonio y Museos of the Junta de Comunidades de Castilla-La Mancha (04-0392-P11) for work conducted in Lo Hueco. The holotype (MDE/CM-616) of *Lohuecosuchus mechinorum* gen et sp nov. described in this paper is deposited in the Paleontological Collection Mechin (Vitrolles, Bouches-du-Rhône, France), affiliated to the Musée des Dinosaures (Espéraza, Aude, France).

### Institutional Abbreviations

**HUE**, Lo Hueco collection, housed at the Museo de las Ciencias de Castilla-La Mancha, Cuenca, Spain; **MDE/CM**, Musée des Dinosaures/Collection Mechin, Espéraza, Aude, France.

### Anatomical Abbreviations

an, angular; ar, articular; bo, basioccipital; bs, basisphenoid; ch, choana; cqg, cranioquadrate groove; cr, coronoid; crt, canthus rostralis; d, dentary; ec, ectopterygoid; ex, exoccipital; f, frontal; fa, foramen aëreum; fcp, foramen caroticum posterius; fe, eustachian foramen; fic, foramen intermandibularis caudalis; fm, foramen magnum; fv, foramen vagi; gf, glenoid fossa; if, incisive foramen; itf, infratemporal fenestra; j, jugal; l, lacrimal; mx, maxilla, n, nasal; na, naris; o, orbit; oc, occipital condyle; or, otic recess; p, parietal; pa, palatine; pf, prefrontal; pm, premaxilla; pmd, premaxillary depression; po, postorbital; pob, postorbital bar; pr, preorbital ridge; pt, pterygoid; ptf, postemporal fenestra; q, quadrate; qj, quadratojugal; qjs, quadratojugal spine; rd, rostral depression; rp, retroarticular process; sa, surangular; so, supraoccipital; sof, suborbital fenestra; sp, splenial; sq, squamosal; stf, supratemporal fenestra; sym, mandibular symphysis; xii, foramen for hypoglossal nerve (XII).

### Systematic Paleontology

Crocodylomorpha [41]

Crocodyliformes [42]

Eusuchia [43]

Allodaposuchidae clade nov.

### Type species

*Allodaposuchus precedens* [21]

### Definition

*Allodaposuchus precedens* and all crocodyliforms more closely related to it than to *Hylaeochampsa vectiana*, *Shamosuchus djadochtaensis*, *Borealosuchus sternbergii*, *Planocrania datangensis*, *Alligator mississippiensis*, *Crocodylus niloticus*, or *Gavialis gangeticus*.

### Included species

*Allodaposuchus precedens* [21]; *Massaliasuchus affuvelensis* [14]; *Musturzabalsuchus buffetauti* [13]; *Arenysuchus gascabadiolorum* [4]; *Allodaposuchus subjuniperus* [5]; *Allodaposuchus palustris* [6]; *Allodaposuchus hulki* [7], *Lohuecosuchus megadontos* sp. nov.; *Lohuecosuchus mechinorum* sp. nov.

### Diagnosis

Member of Eusuchia based on: pterygoids entirely surrounding the margins of the choana; posteriorly oriented external surface of basioccipital ventral to the occipital condyle. Allodaposuchidae is diagnosed by two synapomorphies: shallow fossa at the anteromedial corner of supratemporal fenestra and the tenth alveolus is the largest behind the fourth in the mandibular tooth row. Allodaposuchidae shares with its sister group, Hylaeochampsidae, but not with Crocodylia: cranioquadrate passage laterally opened, without a defined posterior margin of the otic aperture, and without quadrate-squamosal contact posterior to the external auditory aperture; presence of a tubercle in the ventrolateral margin of the paraoccipital processes; absence of an external mandibular fenestra (a feature also found in other derived neosuchians, such as paralligatorids). Allodaposuchidae includes medium-sized crocodyliforms with a brevirostral skull that differ from hylaeochampsids by having a broader cranioquadrate passage represented by a sulcus; less developed tubercle in the ventrolateral margin of the paraoccipital processes; lack of strongly procumbent anterior dentary teeth (shared with Crocodylia); a notch for reception of the fourth dentary, between the premaxilla and the maxilla (shared with *Borealosuchus*, Gavialoidea, Planocraniidae, Crocodyloidea, and *Leidyosuchus*); maxilla excluded from the lower temporal bar (shared with Crocodylia); anterior tip of the frontal constituting a simple acute point (shared with most crcodylians); lacrimal larger than the prefrontal (shared with Gavialoidea, Planocraniidae and Crocodyloidea); skull table with nearly horizontal sides, and significant posterolateral squamosal rami along the paroccipital process (shared with Crocodylia); wide suborbital fenestra (shared with Crocodylia); lack of protuberance in the ventral surface of the quadrate ramus, with an attachment scar for posterior mandibular adductor muscle (shared with Crocodylia); presence of a prominent quadratojugal spine (shared with *Borealosuchus*, Gavialoidea, Planocraniidae and Crocodyloidea); quadrate rami extending beyond the level of the occipital condyle (shared with Crocodylia); upturned dorsal edges of the orbits (shared with several derived crocodylians); dermal bones of the skull roof overhanging the rim of the supratemporal fenestra (shared with caimanines and *Osteolaemus*); quadrate foramen aëreum on dorsal surface of the quadrate (shared with Alligatoroidea); festooned dorsolateral surface of the dentary, describing two concave waves (shared with alligatoroids and crocodyloids).

### Distribution

Late Cretaceous (Campanian and Maastrichtian) of Europe.

*Lohuecosuchus* gen. nov. urn:lsid:zoobank.org:act:101D4DE6-8376-4384-A099-42FD97317935

### Type species

*Lohuecosuchus megadontos* sp. nov. urn:lsid:zoobank.org:act:09A3395F-8E63-4364-A912-24B8E2761749

### Other included species

*Lohuecosuchus mechinorum* sp. nov. urn:lsid:zoobank.org:act:CDB68E47-3F70-4CB8-8654-C2B09B1E8117

### Etymology

The generic name refers to Lo Hueco, the type locality of the type species, and *suchus*, a suffix latinized from the Greek word *souchos*, referring to an Egyptian crocodile-headed god.

### Distribution

Late Cretaceous (late Campanian-early Maastrichtian) of Western Europe.

### Diagnosis

Allodaposuchid characterized by the following autapomorphies: naris wider than long; shallow depressions in the maxillary dorsal surface; well-marked preorbital ridges, and very prominent bosses in the antero-dorsal surface of the maxilla; semi-rectangular anterior palatine process; pterygoid ramus of ectopterygoid straight, and linear posterolateral margin of the suborbital fenestra. *Lohuecosuchus* shares with *Al*. *subjuniperus* and *Ar*. *gascabadiolorum*: presence of a notch between premaxilla and maxilla for the reception of the fourth mandibular tooth; small medial jugal foramen and lingual occlusion between the dentary and maxillary teeth.

*Lohuecosuchus megadontos* sp. nov.

(Figs 2–7)

**Fig 2:**
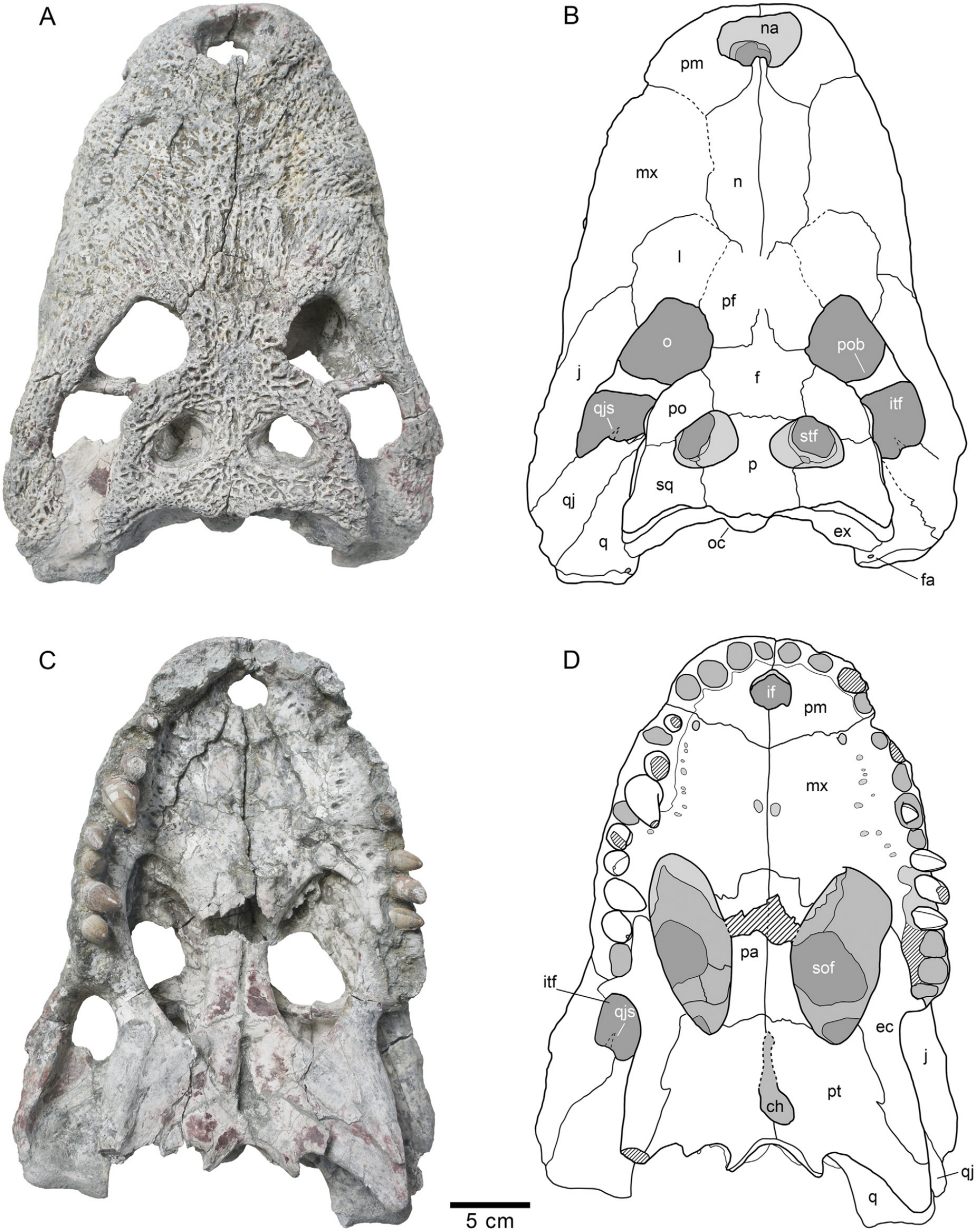
Skull HUE-04498, holotype of *Lohuecosuchus megadontos* gen. et sp. nov. (A, B) Dorsal views. (C, D) Ventral views. A and C, photographs of the specimen. B and D, interpretative drawings. Sutures are figured with plain lines. The dashed lines represent tentatively reconstructed sutures. The lined areas represent broken bones. Specimen from the late Campanian-early Maastrichtian site of Lo Hueco (Cuenca, Spain).

**Fig 3:**
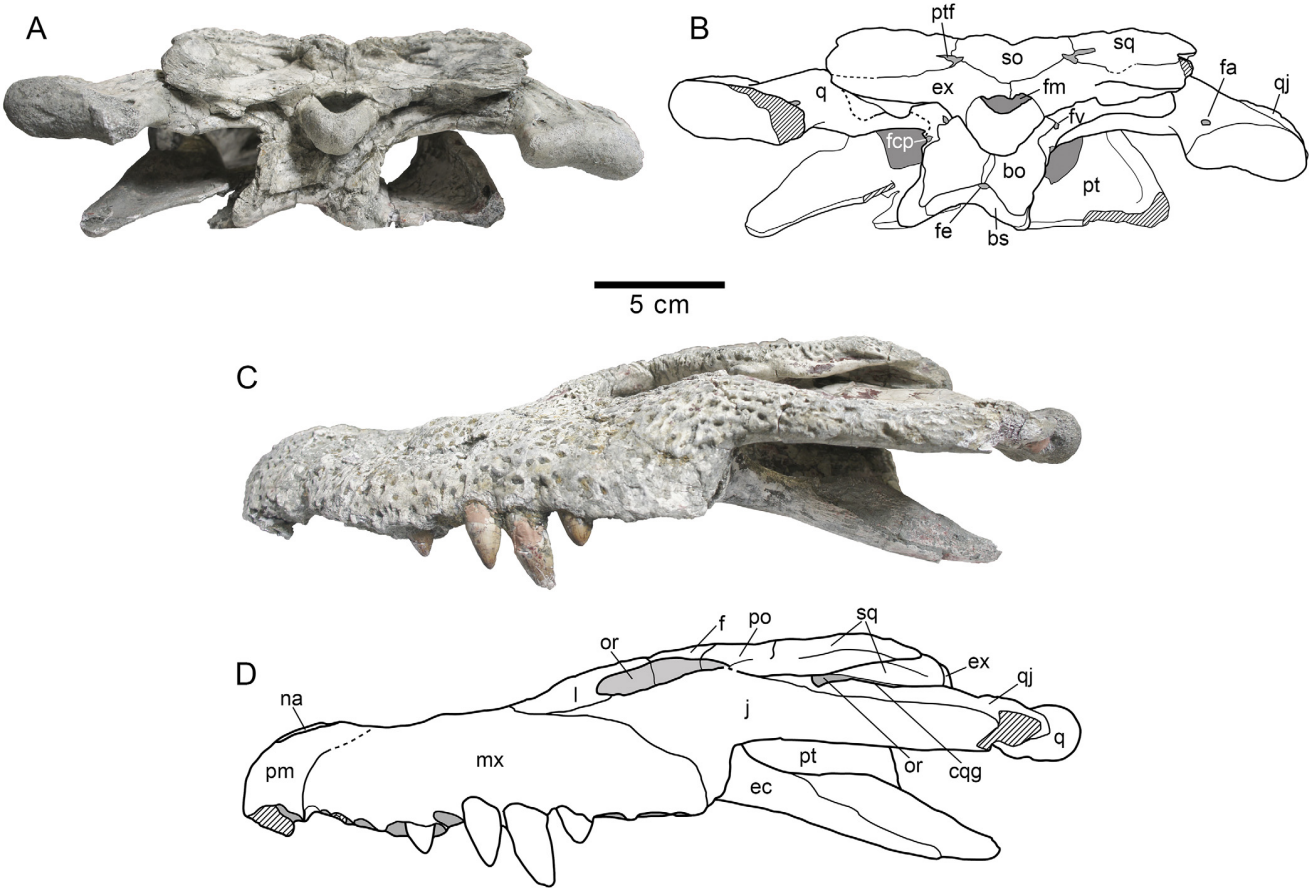
Skull HUE-04498, holotype of *Lohuecosuchus megadontos* gen. et sp. nov. (A, B) Posterior views. (C, D) Lateral views. A and C, photographs of the specimen. B and D, interpretative drawings. Sutures are figured with plain lines. The dashed lines represent tentatively reconstructed sutures. The lined areas represent broken bones. Specimen from the late Campanian-early Maastrichtian site of Lo Hueco (Cuenca, Spain).

**Fig 4:**
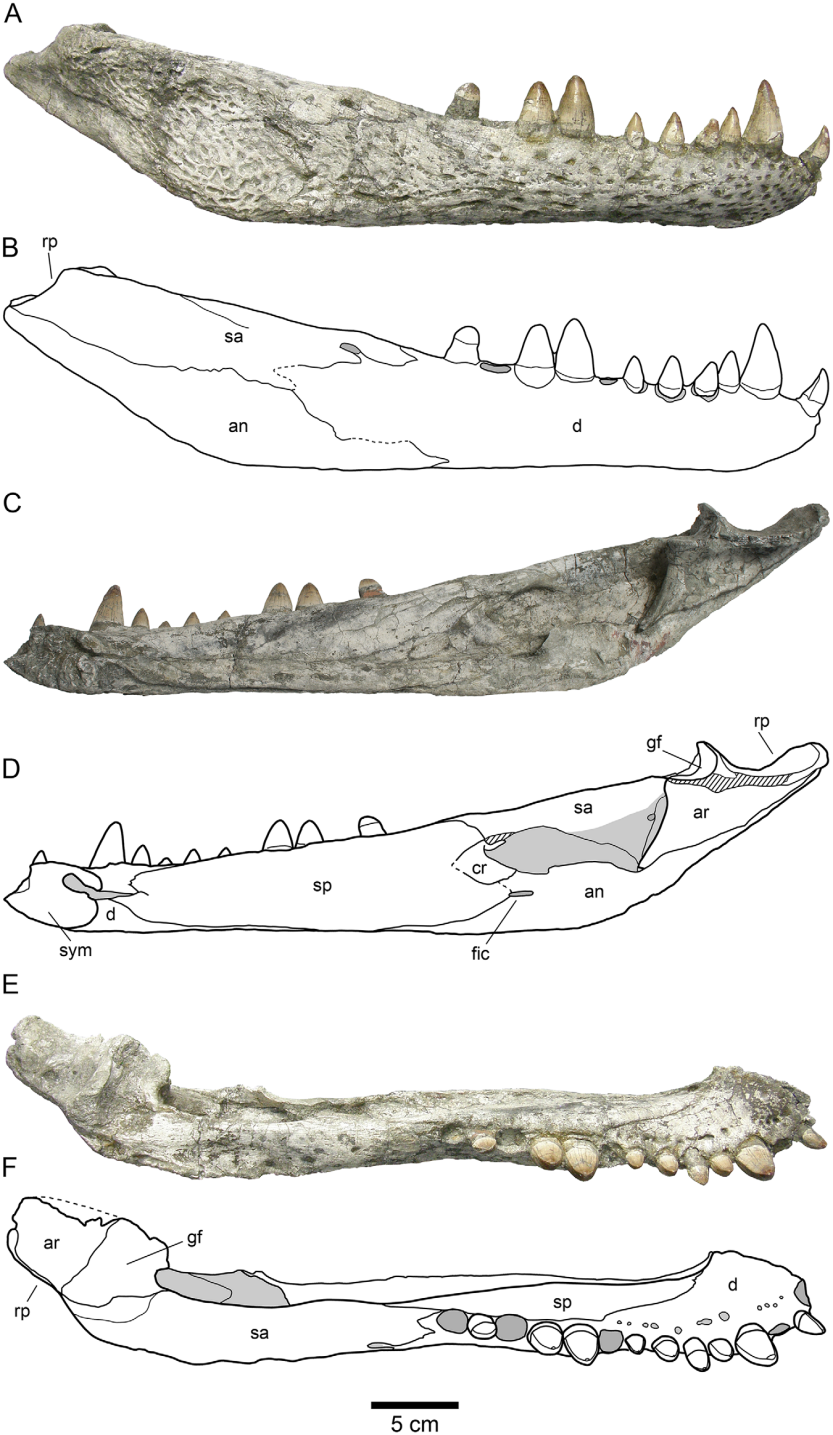
HUE-04498, right jaw of the holotype of *Lohuecosuchus megadontos* gen. et sp. nov. (A, B) Lateral views. (C, D) Medial views. (E, F) Dorsal views. A, C and E, photographs of the specimen. B, D and F, interpretative drawings. Sutures are figured with plain lines. The dashed lines represent tentatively reconstructed sutures. The lined areas represent broken bones. Specimen from the late Campanian-early Maastrichtian site of Lo Hueco (Cuenca, Spain).

**Fig 5:**
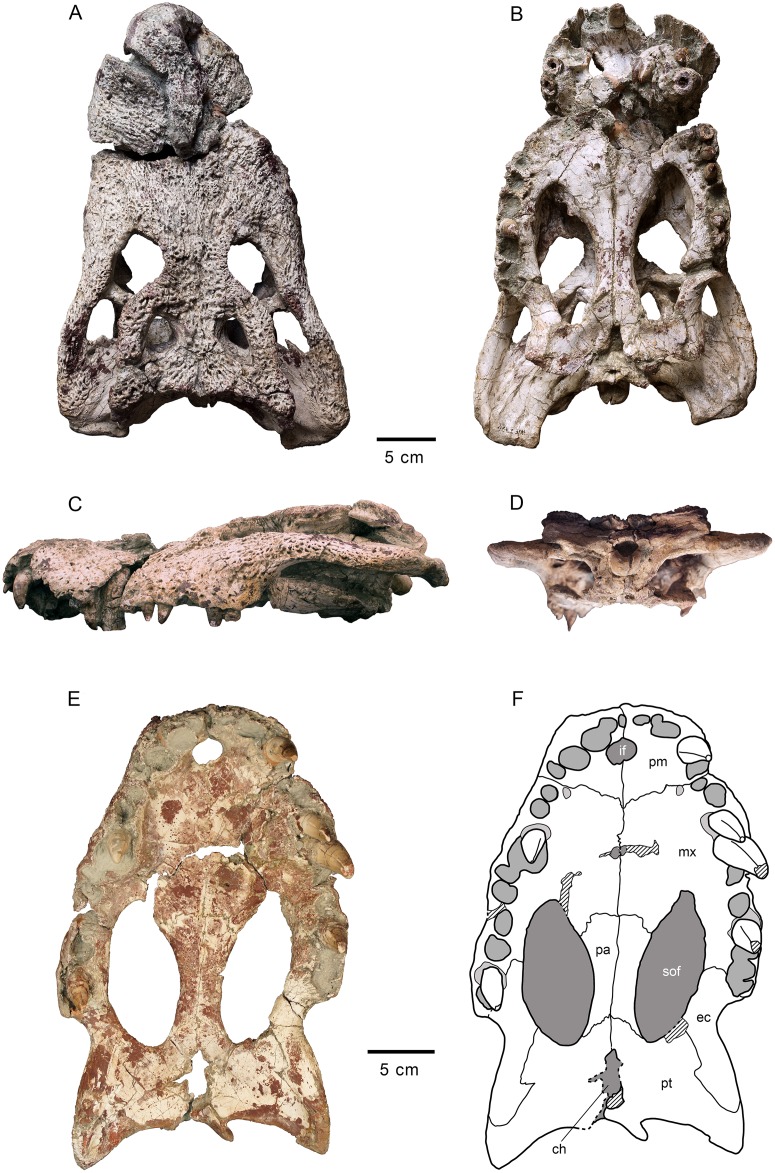
Two of the paratypes of skull *Lohuecosuchus megadontos* gen. et sp. nov. (A–D) HUE-02920, nearly complete skull in dorsal (A), ventral (B), left lateral (C) and posterior (D) views. (E–F) HUE-04263, the palatal region of an incomplete skull, in ventral view. A–E, photographs of the specimens. F, interpretative drawings of HUE-04263. Sutures are figured with plain lines. The dashed lines represent tentatively reconstructed sutures. The lined areas represent broken bones. Specimens from the late Campanian-early Maastrichtian site of Lo Hueco (Cuenca, Spain).

**Fig 6:**
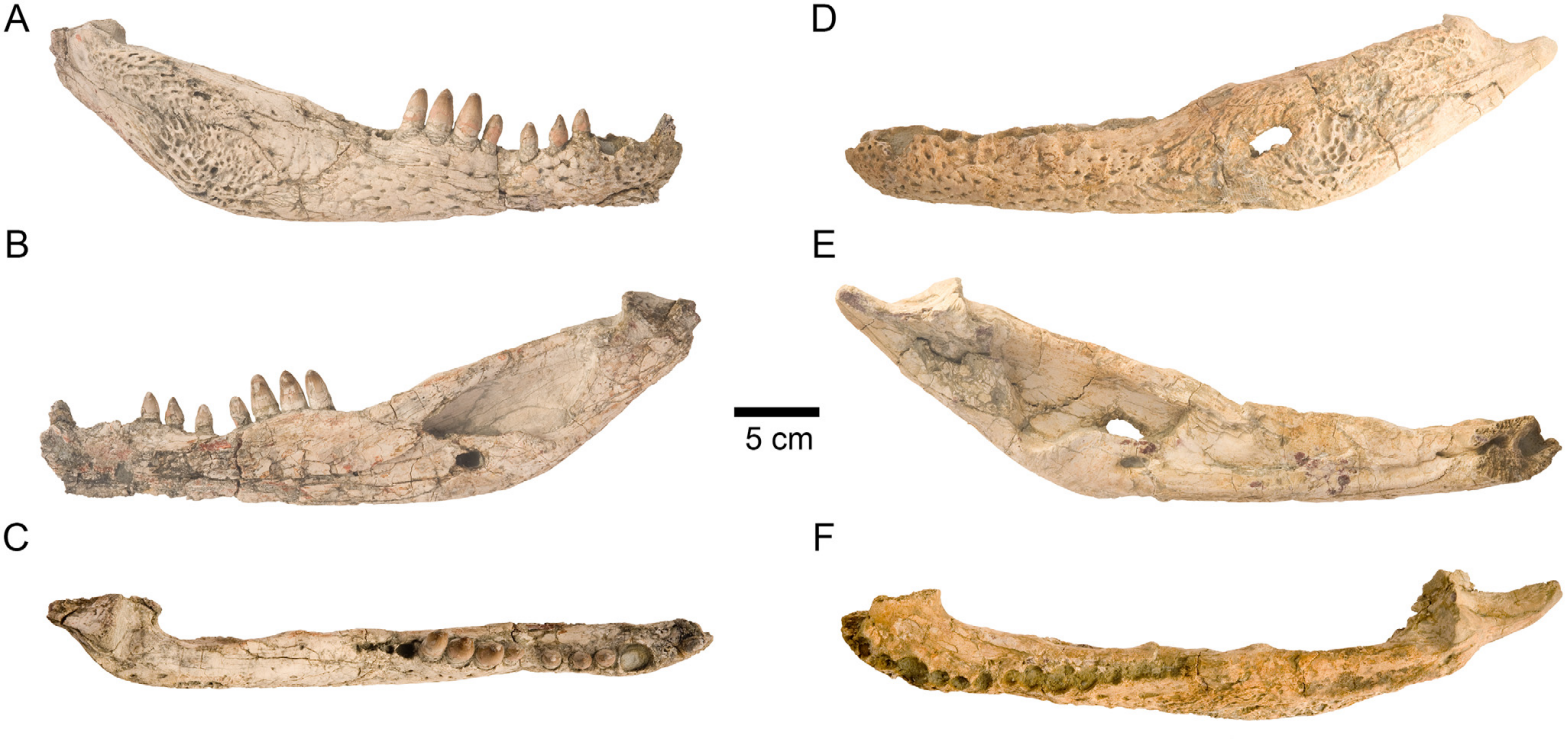
Two of the paratypes of skull *Lohuecosuchus megadontos* gen. et sp. nov. (A–C) HUE-04378, a left lower jaw in labial (A), lingual (B) and dorsal (C) views. (D–F) HUE-05161, a right lower jaw, in labial (D), lingual (E) and dorsal (F) views. Specimens from the late Campanian-early Maastrichtian site of Lo Hueco (Cuenca, Spain).

**Fig 7:**
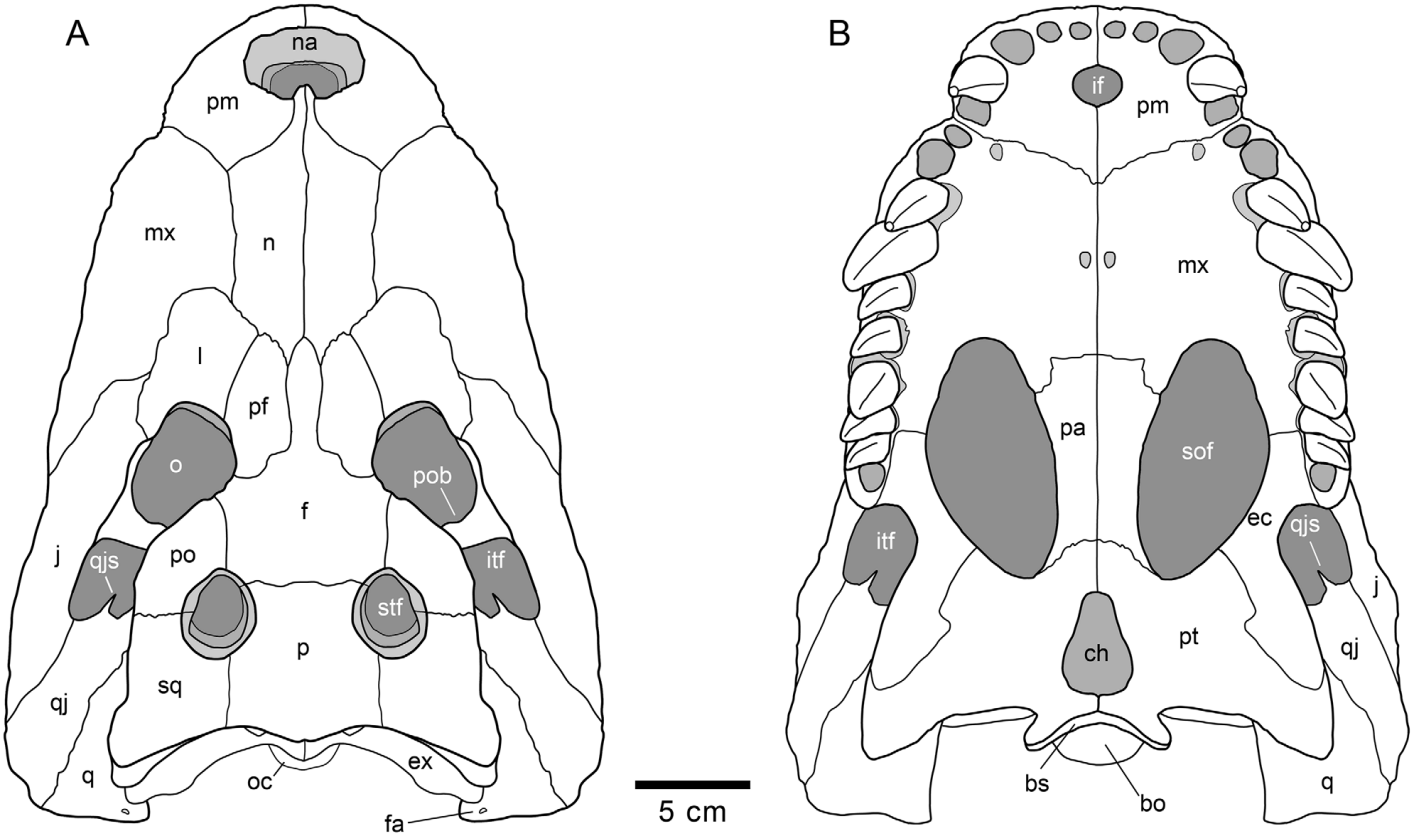
Schematic reconstruction of the skull of *Lohuecosuchus megadontos* gen. et sp. nov. (A) Dorsal view. (B) Ventral view. This reconstruction is based on the holotype (HUE-04498) and the two paratypes corresponding to a partial skull (HUE-04263) and an almost complete skull (HUE-02920).

### Holotype

HUE-04498, a complete skull and right lower jaw (Figs 2–4).

### Paratypes

HUE-02920, a nearly complete skull lacking most of the pterygoids (Fig 5); HUE-04263, the palatal region of an incomplete skull which preserves most of its elements (Fig 5); HUE-04378, a left lower jaw (Fig 6); HUE-05161, a right lower jaw (Fig 6).

### Etymology

The specific name, composed by the Greek words *mega* (meaning big) and *odon* (meaning teeth), refers to the autapomorphic relatively large size of the teeth.

### Type locality and horizon

Lo Hueco fossil site, municipality of Fuentes, Cuenca Province at Castilla-La Mancha (central Spain). Arcas-Fuentes Syncline, southwestern branch of the Iberian Ranges. Margas, Arcillas y Yesos de Villalba de la Sierra Formation, Late Cretaceous (late Campanian-early Maastrichtian) [32, 33] (Fig 1).

### Diagnosis

A species of *Lohuecosuchus* differing from *L*. *mechinorum* by: broad and short rostrum; dental formula with a low number of teeth (four or five premaxillary alveoli, ten or eleven maxillary alveoli, and fourteen or fifteen mandibular alveoli); robust and hypertrophied teeth; closely spaced alveoli, with narrow interalveolar spaces; fourth maxillary alveolus larger than fifth, as with all other members of Allodaposuchidae except *L*. *mechinorum*; premaxillary surface with deep notch lateral to the naris; very prominent canthi rostralii; jugal and ectopterygoid highly vaulted posterior to maxillary toothrow, causing a “jump” in lateral profile; a pair of parasagittal foramina placed in the palatal shelf of the maxillae; jugal-quadratojugal suture placed at the posterior angle of the infratemporal fenestra; palatal shelf of the palatine constitutes a broad and blunt anterior rectangular process that does not extend beyond the anterior limit of the suborbital fenestra; anterior frontal process almost reaches the anterior border of the prefrontals; choana is teardrop-shaped, longer than wide, and posteroventrally oriented; ventral pterygoid-ectopterygoid suture has a zigzag pattern.

### Description

#### Skull

The snout is short, laterally broad and anteriorly rounded (Figs 2, 3 and 5). The maximum length of the skull of the holotype, from the snout tip to the posterior margin of the medial quadrate condyle, is about 385 mm. Its maximum width, at the level of the jugals, is approximately 285 mm. It is therefore a remarkably broad skull relative to its length (Fig 2). The orbits of this taxon are circular and slightly upturned. The supratemporal fenestrae are circular and smaller than the orbits. The infratemporal fenestrae are rectangular and longer than high. The suborbital fenestrae are relatively large and anteroposteriorly elongate. The external surface of the skull is heavily sculpted (Figs 2, 3 and 5).

The premaxillae are wider than long and rounded anteriorly. This pair of bones forms most of the narial margin (Fig 2A and 2B). The external nares are large, circular in profile, and slightly wider than long. They face anterodorsally (Fig 3C). Their margin is smooth, having no ridges, and being situated at the same level as the dorsal surface of the premaxillae. Two deep, anterolaterally directed grooves are situated posterolateral to the external naris. Dorsally, the premaxillo-maxillary suture is straight, slightly bowed, and extends medially from the shallow premaxillo-maxillary notch to the nasal (Fig 2A and 2B). Ventrally, the premaxillo-maxillary suture is posteromedially directed and placed far from the posterior margin of the incisive foramen (Figs 2C–2D and 5E–5F). The incisive foramen is relatively small and circular in outline.

In *Lohuecosuchus*, the number of premaxillary teeth is not the same in all the preserved specimens. Each premaxilla of the holotype bears four alveoli, with the third being the largest (Fig 2C and 2D). The paratypes, HUE-02920 and HUE-04263, bear five premaxillary teeth, with a small second tooth whose alveolus converges with the third (Fig 5B, 5E and 5F). In these specimens, the largest premaxillary alveolus is the fourth.

The maxillae are wide and relatively short. Dorsally, a shallow lateral notch for reception of the fourth dentary tooth separates the premaxilla and the maxilla (Fig 2A and 2B). The dorsal surface of the rostrum presents well-developed rostral canthi. Two shallow depressions extending mediolaterally can be observed on the anterior region of the dorsal surface of the maxilla in the holotype, the left one being more marked. A projected posteriorly sharp process is located between the lacrimals and nasals. In ventral view, there is a large, anterolateral foramen on the palatal shelf of each maxilla near the premaxillo-maxillary suture (Figs 2C–2D and 5E–5F). The maxillae each have ten alveoli closely spaced with narrow interalveolar spaces (Figs 2C–2D, 5B and 5E–5F). However, the left maxilla of the holotype presents eleven alveoli (Fig 2C and 2D). In all specimens the fourth alveolus is the largest. The maxillae constitute the anteromedial corner, and about half of the lateral rim, of the suborbital fenestrae. A parasagittal foramen is located at the mid-length of the medial margin of each maxilla (Figs 2C–2D, 5B and 5E–5F). Nevertheless, none of the foramina for the palatine branch of the cranial nerve V is enlarged.

In *Lohuecosuchus*, the maxillary teeth are extremely robust with respect to the skull proportions (Figs 2C–2D, 5B and 5E–5F). The great development of the teeth is related to the pronounced expansion of the lateral profile, which is especially prominent in the posteriormost portion of the maxillae, affecting the morphology of the ectopterygoid.

Both nasals reach the posterior margin of the naris, separating the premaxillae, but without forming a complete internarial bar (Fig 2A and 2B). The posteriormost portions of the nasals taper gradually and are separate medially for a short sagittal rostral process of the frontal. In turn, each nasal forms a short and acute posterior process between the frontal and prefrontals.

The lacrimals are rectangular and form the anterior margin of the orbits, contacting the jugals posterolaterally, the maxillae anterolaterally, and the nasals and the prefrontals medially. They are longer than the prefrontals (Figs 2A–2B and 5A). Due to preservation, the prefrontal-lacrimal suture is not recognizable in most of the specimens. The dorsal surface of each lacrimal close to the orbit forms a well-marked crest with the prefrontal.

Although the limits of the prefrontals with the lacrimals are not recognized, the sutures of the prefrontals with the rest of the adjacent elements are markedly visible in the holotype and HUE-02920 (Figs 2A–2B and 5A). The prefrontals are wide, with their dorsal surface extending from the midpoint of the medial rims of the orbits to the level of the posteromedial branch of the maxillae. Anteriorly, each prefrontal projects a short and blunt dorsal process. Prefrontal pillars are not well preserved in any of the specimens due to the collapse of the skull table. As preserved, they are columnar parasagittal structures that dorsally begin at the medial border of the prefrontal, close to the frontal suture. The pillars have well-developed medial processes which contact the sagittal plane. Laterally, the prefrontal pillars form a wide lamina that extends transversally the complete ventral side of the prefrontal body. In anterior view, this lamina forms a wide concave wall under the anteromedial corner of the orbits.

The frontal bears a long and slender anterior process separating the prefrontals and extending between the nasals for a short distance (Figs 2A–2B and 5A). This anterior process does not extend beyond the anterior margins of the prefrontals. The margins of the orbits are slightly upturned, and the dorsal surface of the frontal between the orbits is shallowly concave (Figs 3C and 5C). The frontal forms the posteromedial corners of the orbits, and posteriorly, the anteromedial margins of the supratemporal fenestra.

The parietal is flat, and its dorsal surface is flush with the medial margins of the supratemporal fenestrae (Figs 2A–2B and 5A). Posteriorly, it bears a dorsal midline depression. Its suture with the frontal is almost linear and intersects the supratemporal fenestrae.

Dorsally, the postorbitals are crescentic in shape (Figs 2A–2B and 5A). A descending process is inset on the ventral side and forms the dorsal portion of the postorbital bar. The postorbital portion of the postorbital bar is subtriangular in cross-section and represents half of its length. The postorbitals form the posterior rims of the orbits and the anterolateral corners of the supratemporal fenestrae. The postorbital-jugal sutures are not clearly visible.

**Fig 8:**
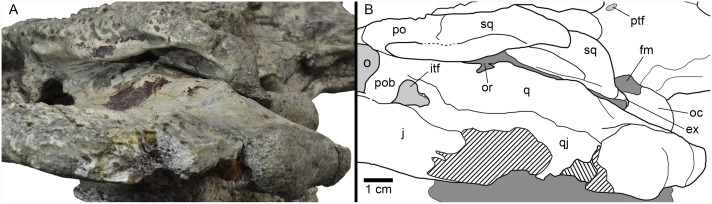
Left posterodorsolateral view of the otic region of HUE-04498, holotype of *Lohuecosuchus megadontos* gen. et sp. nov. (A) Photograph of the specimen. (B) Interpretative drawing.

The squamosals have a flat dorsal surface (Figs 2A–2B and 5A). The posterolateral process slopes ventrally, forming the anterolateral wall of the paraoccipital process. In dorsal view, the posterolateral tip of the squamosals is ornamented. This tip is weakly developed and is placed almost over the paroccipital processes that, in this view are slightly posterolaterally projected. A notch, visible in lateral and occipital view, separates the ornamented dorsal surface of the skull table from the unornamented paroccipital process (Figs 3A–3B and 5D). In lateral view, an anterior process of the squamosals underlies the postorbitals to reach the postorbital bars (Figs 3C–3D and 8A–8B). This anterior projection forms a prominent boss under the postorbitals, as can be observed in dorsal view (Figs 2A–2B and 5A). The lateral squamosal rims and groove for insertion of the ear flap musculature are longitudinally developed. The dorsal rim is shorter and restricted to the rear part. The ventral rim is more prominent and overhangs the otic recess. The squamosals form most of the dorsal outline of the otic aperture (Fig 8A and 8B). In occipital view, the squamosals constitute the lateral half of a prominent depression on the occipital surface, lateral to the postemporal fenestra (Figs 3A–3B and 5D).

The cranioquadrate passage is an open groove extending from the otic aperture to the rear of the otic recess (Figs 3A–3B, 5D and 8A–8B). It does not form a tube enclosed laterally by the squamosal and quadrate. Details within the groove are difficult to recognize but, probably, the ventral surface is formed by the exoccipital. Therefore, the absence of contact between the squamosal and quadrate posterior to the otic aperture is interpreted.

The jugals form the posteroventral margin of the orbit (Figs 2A–2B and 5A). The anterior ramus of the jugal extends to the eighth maxillary alveolus and tapers anteriorly. The ventral outline has a marked concavity behind the tooth row corresponding with the size of the posterior teeth and the morphology of the ectopterygoid. The posterior ramus of the jugals is robust and slightly compressed laterally, but not laminar. The postorbital bars are inset medially on the jugals, being slender. The basal section of the postorbital bars is anteroposteriorly compressed. The jugals bear a relatively large foramen on the internal surface, posterior to the base of the postorbital bar.

The quadratojugals form the posterior margin of the infratemporal fenestra, contacting the postorbitals, and preventing the contact of the quadrate with the margin of the fenestra (Figs 2A–2B, 5A and 8A–8B). A large spine is present on the right quadratojugal of HUE-02920 (Fig 5A). These spines are broken in the holotype. The quadratojugal-jugal suture lies at the posteroventral corner of the infratemporal fenestra. The quadratojugals extend posteriorly lateral to the quadrate, but does not participate in the condyle.

The dorsal surface of the quadrates is smooth (Figs 2A–2B, 5A and 8A–8B). The foramen aëreum is located on the dorsomedial surface and opens at the end of a low crest. The quadrates form the posteroventral margin of the external otic aperture. As described above, the quadrates do not contact the squamosal in the rear part of the otic notch, creating a laterally open cranioquadrate passage. In ventral view, two well-marked parallel and sinusoidal crests that extend along the main axes of the quadrates are identified. These crests define a groove that ends at the foramen for cranial nerve V (Figs 2C–2D, 5B and 5E–5F). One of these, roughly similar to the “crest A” sensu Iordansky (1973) [44], extends laterally parallel to the suture with the quadratojugal, and medially very close to the ventromedial edge of the supratemporal fenestra. The other one possibly corresponds to the “crest B” sensu Iordansky (1973) [44]. It extends near to the medial border of the quadrates. The quadrate condyles are both wider than high (Figs 3A–3B and 5D). The main axis of the articular surfaces of the condyles is almost horizontal, with a slight ventral deflection on the medial hemicondyle. The medial hemicondyle is smaller than its lateral counterpart. The quadrates form the posterior wall of the ventral part of the supratemporal fenestra, contacting the ventral outline of the temporal canal on the posterior fenestral wall.

The palatines are joined along the midline (Figs 2C–2D, 5B and 5E–5F). Their palatal surface is relatively wide and their lateral outlines are nearly straight, except on their anterior portion, where they are rostro-laterally widened. Anteriorly, they form a broad and blunt rectangular process between the maxillae that does not extend beyond the anterior margin of the suborbital fenestrae. The palatines are short and, although they form most of the medial outline of the suborbital fenestrae, they do not reach the anteromedial or posteromedial corners of these fenestrae. The palatine-pterygoid suture is slightly curved and projects anteriorly (Figs 2C–2D, 5B and 5E–5F).

The ectopterygoids are thick and robust. They contact the posteromedial area of the maxillae, forming the medial wall of the posteriormost two, and sometimes three, maxillary alveoli (Figs 2C–2D, 5B and 5E–5F). The anterior process of the ectopterygoids tapers against the maxilla and does not form a palatal shelf. In the rear part of the hypertrophied tooth row, the ectopterygoids are slightly projected laterally, partially embracing the posterior part of the last alveolus. A short ascending process forms part of the medial side of the postorbital bars and contacts with the descending process of the postorbitals. Each ectopterygoid forms the posterolateral margins of the suborbital fenestrae (Figs 2C–2D, 5B and 5E–5F).

The ventral surface of the pterygoids is almost flat, these bones being slightly depressed sagittally. The choana is completely surrounded by the pterygoids (Figs 2C–2D, 5B and 5E–5F). The choana is teardrop in shape, longer than wide and posteroventrally oriented. There is no septum dividing the choana. A thin sagittaly elongate groove between the pterygoids extends anteriorly from the choana. Laterally, each pterygoid contacts the ectopterygoid. The ectopterygoids, which lie on the ventrolateral pterygoid surface, do not extend beyond the posterior tip of the pterygoid wing. In ventral view, the ectopterygoid-pterygoid sutures show a zigzag pattern. They contact the posterolateral corner of the suborbital fenestrae anteriorly (Figs 2C–2D and 5E–5F). Posteriorly, the pterygoid wings do not reach the length of the quadrate hemicondyles. However, the pterygoid wings surpass the level of the posterior margin of the posteromedial pterygoid processes.

The two most complete preserved skulls, HUE-04498 and HUE-02920, are slightly compressed dorsoventrally in the rear part, and this is especially evident in the area of the lateroventral wall of the braincase (Figs 3A–3B and 5D). Therefore, a detailed description of this area is not possible. The laterosphenoids are almost complete and distorted, but their dorsal processes are triangular, ending as incompletely preserved capitate processes, probably being anteroposteriorly expanded. The laterosphenoid-quadrate sutures are vertical below the supratemporal fenestra. These sutures reach the dorsal borders of the trigeminal foramen, strongly deformed or unexposed in all the specimens.

The supraoccipital is wider than high in occipital view. It presents a subtriangular outline (Figs 3A–3B and 5D). There is a well-marked nuchal crest along its midline, which does not reach the exoccipital. The postemporal fenestrae are dorsoventrally flattened, but it is possible to recognize their ventral margin, constituted by well-developed external occipital protuberances. The lateral borders of the supraoccipital form the dorsomedial margin of well-marked depressions located lateral to postemporal fenestrae (Figs 3A–3B and 5D). It is not evident whether the supraoccipital takes part of the dorsal surface of the skull table because it is not possible to recognize the parieto-supraoccipital suture. There is a sagittal depression at the posterior outline of the cranial table, affecting the rear part of the parietal and the dorsal part of the supraoccipital (Figs 2A–2B and 5A).

The exoccipitals form the dorsolateral margin of the foramen magnum, which is crushed dorsoventrally in all available specimens (Figs 3A–3B and 5D). The exoccipitals meet above this opening. The dorsal half of the exoccipitals also forms the posterior surface of the paraoccipital processes, which exceeds the level of the medial margin of the quadrate condyles. The dorsal surface of the exoccipitals appears depressed in contact with the squamosals. The ventral region of the exoccipitals laterally contacts the quadrates. The ventrolateral margins of this region bear the foramen caroticum posterius and, lateral to the occipital condyle, the medial margin the foramen vagi (Figs 3A–3B and 5D). Due to the preservation, the foramina for hypoglossal nerve (XII) cannot be identified. In the otic region, the exoccipitals form the inner wall of the cranioquadrate grooves (Fig 8A and 8B), and connect dorsally with the quadrate and ventrally with the squamosal, separating them.

The basisphenoid is well exposed in occipital view, below the ventral margin of the basioccipital, and shows a vertical occipital surface (Figs 3A–3B and 5D). The contact area with the pterygoids is not clear. The basisphenoid forms the ventromedial margin of the crushed medial eustachian foramen. Therefore, the lateral eustachian openings cannot be recognized. In lateral view, collapse of the braincase obscures contact areas between the basisphenoid and adjacent elements (Figs 3A–3B and 5D).

The occipital region of the basioccipital forms most of the occipital condyle (Figs 3A–3B and 5D). The occipital condyle does not bear a well-developed neck. Its dorsal wall forms the ventral margin of the foramen magnum. Sagittally, on its ventral half, the basioccipital bears a well-marked crest, extending longitudinally from the posteroventral end of the occipital condyle to the ventral margin of the basioccipital. This ventral margin is wide and has a sagittal concavity at the end of the ridge, between both basioccipital tubera. The basioccipital also forms the dorsolateral margin of the medial eustachian foramen.

#### Lower jaw

The maximum length of the lower jaw in the holotype, from the tip of the retroarticular process to the anterior margin of dentary, is about 470 mm. Its maximum height, from the ventral margin of the dentary to the posterior tip of the glenoid fossa, is approximately 110 mm (Fig 4A–4F).

The dentary is a long, tall and robust element that constitutes more than half of the length of the lower jaw. The dentary of the holotype has fourteen closely spaced and circular alveoli, nine of which bear complete teeth (Fig 4A–4F). HUE-04378 has fifteen alveoli (Fig 6D–6F) and HUE-05161 has fourteen, with eight complete teeth (Fig 6A–6C). However, in the latter specimen the third alveolus has been reabsorbed. The fourth, tenth and eleventh teeth are the largest in the holotype, whereas the fourth, ninth and tenth are the largest teeth in both HUE-04378 and HUE-05161 (Figs 4A–4F and 6A–6C). Considering the reabsortion indicated above, the largest dentary alveolus caudal to the fourth is the tenth. In all specimens there is a diastema between the seventh and eighth alveoli. The tooth row is laterally projected, especially in its anterior area. The dentary symphysis is robust and extends posteriorly to the level of the fourth alveolus. The lateral surface of the dentary is heavily ornamented, except in its posterior region. An external mandibular fenestra is absent in all specimens (Figs 4A–4B, 6A and 6D).

The splenial covers the medial surface of the mandibular ramus. It is smooth and almost flat (Figs 4C–4D, 6B and 6E). It is imperforate, with no foramina on its surface. Anteriorly, the splenial extends up to the level of the fourth dentary alveolus, near the symphysis, but do not form part of it. At its anterior border, delimiting the Meckelian groove, the ventral process is longer than the dorsal. Posterodorsally, the splenial contacts the last five alveoli, beginning with the tenth and forming the medial wall of the posteriormost three alveoli (Figs 4E–4F, 6C and 6F). The rear portion of the splenial forms the anterior border of the foramen intermandibularis caudalis (Figs 4C–4D, 6B and 6E).

The right coronoid is preserved in articulation on the holotype, but it is broken anteriorly and, therefore, the foramen intermandibularis medius cannot be observed (Fig 4C and 4D). The coronoid is crescent-shaped. In this specimen, its caudal margin is damaged and presents a small notch in its middle area. The posterior border of the coronoid forms the anterodorsal margin of the mandibular fossa.

The angular forms near the posteroventral portion of the jaw, extending from close to the posteriormost tip of the retroarticular process to an acute process on the ventral surface of the dentary, at approximately the level of the penultimate dentary alveolus (Figs 4A–4D, 6A–6B and 6D–6E). In labial view, its anterior half is strongly ornamented with large shallow depressions, but its rear half is smooth and lacks foramina, as well as their lingual, ventral and back surfaces (Figs 4A–4B, 6A and 6D). In lateral view, a low pronounced ridge extends parallel to the suture with the dentary. In its lingual surface, angular forms most of the foramen intermandibularis caudalis. The medial ascending lamina does not extend anterior to the coronoid (Figs 4C–4D, 6B and 6E).

The surangular is a long and robust bone (Figs 4A–4F and 6A–6F). It forms almost half of the dorsal length of the mandible. It presents a flat, broad and anteroposteriorly elongate shelf on its dorsal surface (Figs 4E–4F, 6C and 6F). The anterior end of the surangular bears two processes, with the dorsal process being longer than its ventral counterpart (Figs 4A–4B, 6A and 6D). An enlarged foramen, bound by the anterior processes, is present at the anterior end. Dorsally, a process contacts the posteromedial margin of the last alveolus and separates the splenial and dentary. Posteriorly, the surangular extends to the posterior end of the retroarticular process and contacts the lateral wall of the glenoid fossa (Figs 4E–4F, 6C and 6F). Except in the anterior and posterior processes, the labial face of the surangular is heavily ornamented (Figs 4A–4B, 6A and 6D).

The articular forms the glenoid fossa and retroarticular process (Figs 4C–4F, 6B–6C and 6E–6F). The retroarticular process is long and wide and posterodorsally oriented, with a strongly concave dorsal surface. The foramen aëreum is obscured and its position on the retroarticular process is unclear. The lingual surface of the articular is concave and tapers ventrally to an anterior process, forming the posterior wall of the mandibular fossa (Figs 4C–4D, 6B and 6E).

#### Dentition

The dental formula of *Lohuecosuchus megadontos* includes four or five premaxillary alveoli, ten or eleven maxillary alveoli and fourteen or fifteen alveoli in the dentary (Figs 2C–2D, 3C–3D, 4A–4F, 5B–5C, 5E–5F and 6A–6F). The pattern of dentary-maxillary occlusion is lingual. Variations from anterior to posterior tooth morphology are similar in the lower and upper tooth rows. Preserved maxillary and dentary teeth in the holotype are conical. Most teeth are pointed, but robust. All maxillary and mandibular teeth have a flat lingual surface. Two longitudinal well-marked and smooth carinae are developed from the base to the apex, on the mesial and distal surfaces. Both carinae do not show neither denticles nor crenulations. The crowns have a D shaped cross-section with the mesial and distal carinae lingually displaced. The labial surface is thus concave, and the apex is lingually directed. The enamel has neither ridges nor ornamentation. Relative size of all teeth of the series is larger than those for the same tooth positions in other eusuchians. As discussed above, there are some differences in the size of the teeth, probably related to the rostral festooning. Besides that, the last preserved mandibular tooth, which is located on the penultimate alveoli, has a short, wide and blunt molariform crown (Fig 4A–4F). The exact number of this type of tooth in the dentary (up to three) or its presence or absent in the maxilla (in which the presence should be restricted to the last alveolus) cannot be confirmed, but this morphotype is not uncommon as isolated elements in the fossil site.

*Lohuecosuchus mechinorum* sp. nov.

(Figs 9 and 10)

**Fig 9:**
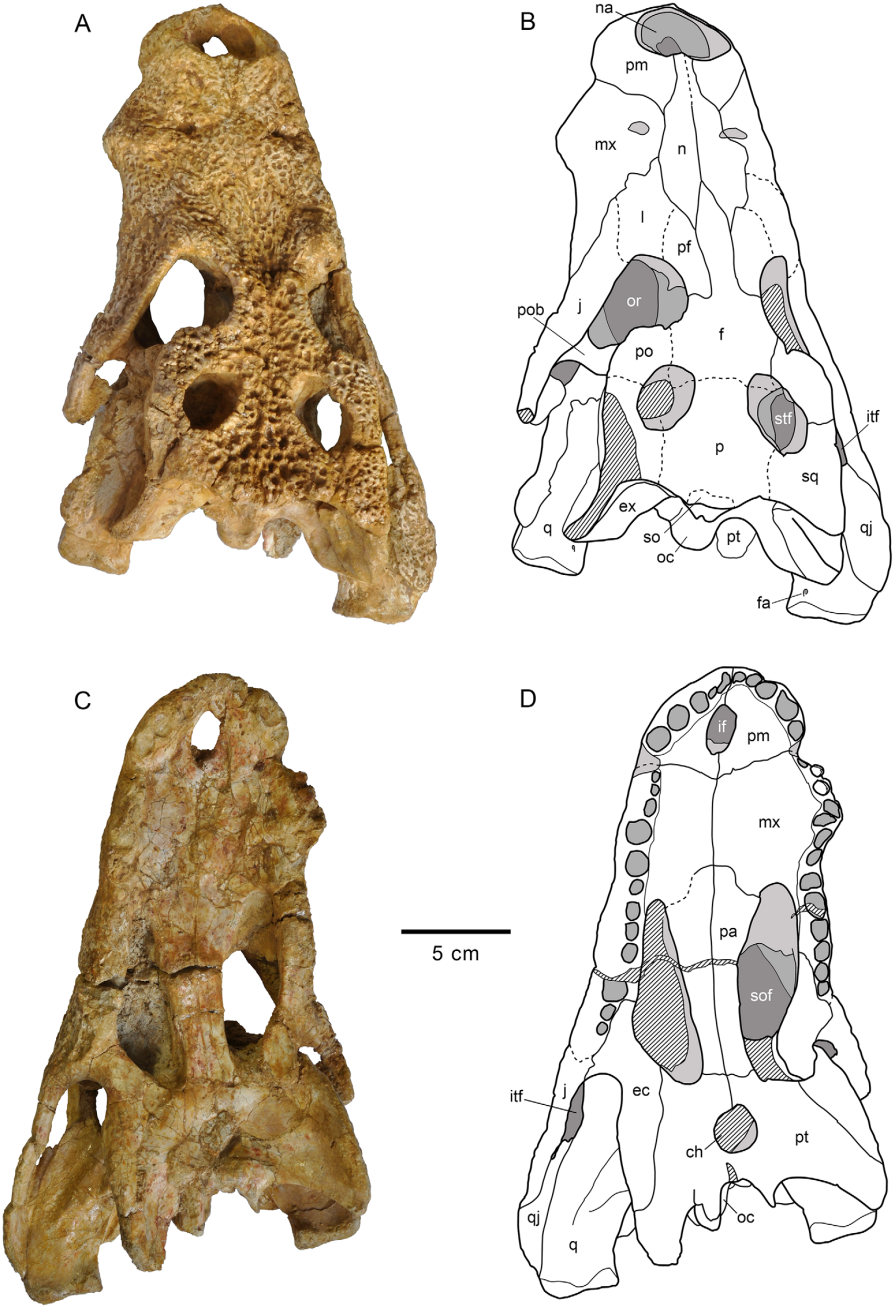
Skull MDE/CM-616, holotype of *Lohuecosuchus mechinorum* gen. et sp. nov. (A, B) Dorsal views. (C, D) Ventral views. A and C, photographs of the specimen. B and D, interpretative drawings. Sutures are figured with plain lines. The dashed lines represent tentatively reconstructed sutures. The lined areas represent broken bones. Specimen from the late Campanian-early Maastrichtian site of Fox-Amphoux (Var, France).

**Fig 10:**
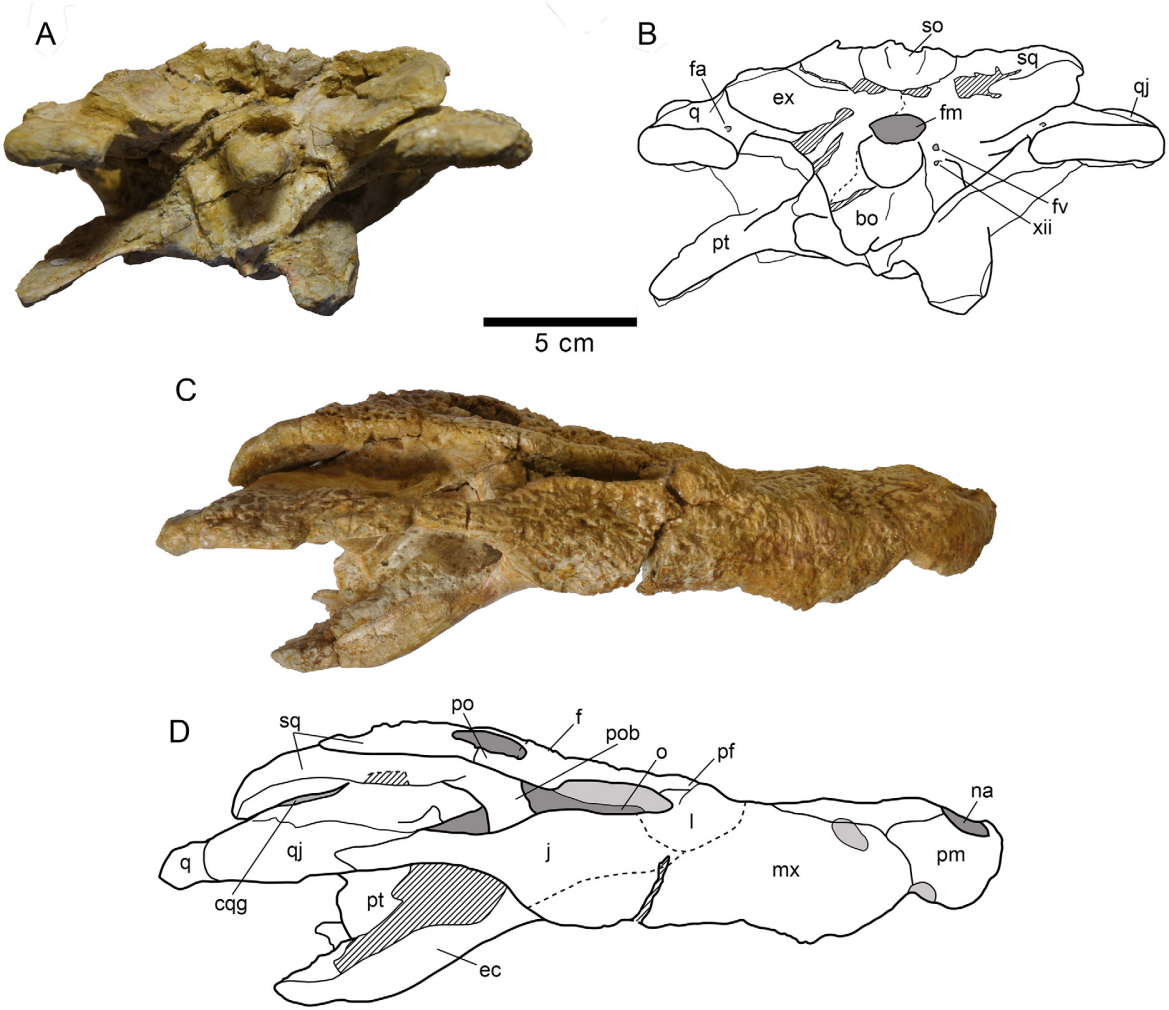
Skull MDE/CM-616, holotype of *Lohuecosuchus mechinorum* gen. et sp. nov. (A, B) Posterior views. (C, D) Lateral views. A and C, photographs of the specimen. B and D, interpretative drawings. Sutures are figured with plain lines. The dashed lines represent tentatively reconstructed sutures. The lined areas represent broken bones. Specimen from the late Campanian-early Maastrichtian site of Fox-Amphoux (Var, France).

### Holotype

MDE/CM-616, a complete skull (Figs 9 and 10).

### Etymology

In honor of the discoverers of the holotype, Patrick and Annie Mechin.

### Type locality and horizon

Fox-Amphoux fossil site, Department of Var, southeastern France. Grès à Reptiles Formation, Late Cretaceous (late Campanian-early Maastrichtian) [22, 45].

### Diagnosis

A species of *Lohuecosuchus* differing from *Lohuecosuchus megadontos* by: more elongated rostrum; dental formula with five premaxillary and twelve or thirteen maxillary alveoli; teeth similar to those of all the members of Allodaposuchidae except *Lohuecosuchus megadontos*; interalveolar spaces similar to that of all the members of Allodaposuchidae except *Lohuecosuchus megadontos*; fourth maxillary alveolus largest, but fifth alveolus also very large and only slightly smaller than the fourth; premaxillary surface without deep notch lateral to the naris; lack of canthi rostralii; absence of a marked jump posterior to the tooth row; lack of parasagittal foramina in the palatal shelf of the maxillae; jugal-quadratojugal suture placed lateral to the posterior angle of the infratemporal fenestra; palatal shelf of the palatine constituting a broad and blunt anterior rectangular process, extending beyond the anterior limit of the suborbital fenestra; anterior frontal process extending beyond the anterior margin of the prefrontals; rounded and posteroventrally oriented choana; straight ventral pterygoid-ectopterygoid suture.

### Description

#### Skull

The skull is brevirostral and unidirectionally deformed (Figs 9A–9D and 10A–10D). The maximum length of the skull, from the snout tip to the posterior margin of the lateral quadrate condyle, is about 290 mm. Its maximum width, at the level of the quadratojugals, is approximately 180 mm. Its outline is markedly festooned, especially in the left side of its anterior maxillary margin, probably due to deformation. The orbits are rounded and their edges are upturned relative to the dorsal surface (Fig 9A and 9B). Anteroposteriorly, the supratemporal fenestrae are slightly oval and smaller than the orbits. The infratemporal fenestrae are narrow, probably as a result of the deformation. The suborbital fenestrae are anteroposteriorly elongated and very narrow, especially in the rostral region (Fig 9C and 9D).

The premaxillae are wider than long and anteriorly rounded (Fig 9A–9D). Premaxillae form much of the margin of the naris, which is large, wider than long and anterodorsally projected (Figs 9A–9B and 10C–10D). In dorsal view, the premaxillo-maxillary suture is slightly curved. It extends from the notch for reception of the fourth mandibular tooth to the lateral suture of the nasal, forming a short posterior premaxillary process that reaches the level of the third maxillary alveolus (Fig 9A and 9B). In palatal view, the premaxillo-maxillary suture is straight, being posteromedially directed near the sagittal axis (Fig 9C and 9D). It is located far from the posterior margin of the incisive foramen, which is small and anteroposteriorly oval, probably due to deformation. Each premaxillary branch has five alveoli, the first two being the smallest of the series and the fourth the largest.

The maxillae are almost twice as long as wide (Fig 9A–9D). In dorsal view, they present an antero-lateral notch for the reception of the fourth mandibular tooth, which bears the suture that separates the premaxilla and maxilla (Fig 9A and 9B). Two shallow depressions, extending mediolaterally on the anteromedial area of the dorsal surface of the maxilla, are identified. They are extending up to the fourth maxillary alveolus. According to Martin (2010) [22], these depressions, which are symmetrical in both maxillary branches, do not have a post mortem origin. Posteriorly, there is a short pointed process in the left maxillary branch that gets inside the lacrimal. This structure is not observed in the right maxillary branch because the suture is unclear. In ventral view, no foramina for palatine ramus of cranial nerve V are observed (Fig 9C and 9D). The palatal surface is elevated relative to the tooth row on the right maxilla. However it is not possible to observe this elevation in the left maxilla due to deformation of this element. The right maxilla preserves twelve alveoli, but a crack may be obscuring a thirteenth (Fig 9C and 9D). On the left maxilla there are thirteen alveoli. The second and third teeth are preserved. The alveoli are round, the first three being the smallest, and the fourth being the largest in the series. The fifth maxillary alveolus is slightly smaller than the fourth and much larger than successive alveoli. The maxillae form the anterior and half of the lateral margins of the suborbital fenestrae, but are not involved in the medial margins of these fenestrae (Fig 9C and 9D).

Dorsally, the nasals are wide and laterally expanded in their posterior half, and narrow in their more anterior region, where they contact the posterior margin of the naris. They separate both premaxillae medially (Fig 9A and 9B). In their rear region, the nasals are thinner and are separated by a sharp anterior process of the frontal, forming two posterior nasal processes between the frontal and prefrontals.

The lacrimals are rectangular. The posterolateral sutures, in the area of contact with the jugals, are not well-preserved (Fig 9A and 9B). The lacrimals are longer than the prefrontals. The lacrimals form the rostral region of the orbits and constitute the distal portion of the dorsal preorbital ridges.

The prefrontals are elongated and form the anteromedial margins of the orbits (Fig 9A and 9B). Both are separated by a long and narrow anterior process of the frontal. This anterior process extends beyond the anterior margin of the prefrontals. At the middle region of the frontal, the prefrontals are mostly elevated on the dorsal surface of the rostrum, forming a marked ridge ahead of the anteromedial margin of the orbit. A small foramen is observed in the prefrontal wall of the orbits.

The frontal is highly ornamented, and this hinders detailed observation of sutures with adjacent elements (Fig 9A and 9B). This bone constitutes the anteromedial margins of the supratemporal fenestrae and the slightly elevated posteromedial margins of the orbits. A long and narrow anterior process extends beyond the anterior margin of the prefrontals in its more rostral region. In dorsal view, the posterior contact of the frontal with the parietal cannot be appreciated, although it is possible to observe the sutures between both elements in the medial wall of the supratemporal fenestrae (Fig 9A and 9B). The dorsal surface of the frontal is slightly depressed.

The parietal presents large and deep depressions constituting the ornamentation of dorsal surface (Fig 9A and 9B). Its dorsal surface is slightly depressed respect to the rest of the skull table. The limits of this bone with the squamosals and the frontal are not clear. Meanwhile, the contact region of the parietal with the supraoccipital bears a notch at its posterior margin with two small projections on both sides. The parietal forms most of the medial margins of the supratemporal fenestrae, being slightly projected above them.

In dorsal view, the postorbitals are longer than wide. Each is approximately half the length of the squamosal (Fig 9A and 9B). The postorbitals form the anterolateral margins of the supratemporal fenestrae. The sutures of these bones with the squamosals and the frontal can only be observed in the right side. The postorbital bars are massive and are inset into the anteroventral region of the postorbitals (Figs 9A–9B and 10C–10D).

The right squamosal is almost complete, whereas the left one is posterolaterally broken (Fig 9A and 9B). The dorsal surface of the squamosals is flat and ornamented. The suture of the squamosals with the parietal cannot be observed. Posteriorly, the squamosals present a narrow, elongated and ventrally oriented projection. The posterior margins of the squamosals slightly overhang the occipital areas. In their lateral surface, there is a shallow groove for insertion of the ear flap musculature, whose lower margin is projected anteriorly, contacting with the postorbital bar (Fig 10C and 10D). The squamosals constitute the largest part of the roof of the otic aperture. Their posterior region does not contact the quadrates.

The left jugal is incomplete on its rear region (Fig 9A and 9B). The jugals constitute the lateral margin of the infratemporal fenestrae and the lateral margins of the orbits, forming a thick border. Anteriorly, the jugals project an acute rostral process that reaches the eighth maxillary alveolus. In dorsal view, their posterior development is short and does not exceed the limit of the posterior margins of squamosals (Fig 9A and 9B). A small medial foramen is developed ahead of the basis of the postorbital bars.

Both quadratojugals are preserved. The right quadratojugal is almost complete, but the left one is broken in its lateral region (Fig 9A–9D). These elements are long and ornamented, and form the posterior margins of the infratemporal fenestrae. The quadratojugal projects a long anterior process excluding the quadrate from the fenestra. The suture between the quadratojugals and the jugals is slightly anterior relative to the rear angles of the infratemporal fenestrae. The quadratojugal spines are not preserved. Posteriorly, neither quadratojugal reaches the posterior end of the quadrate ramus. They do not participate in the mandibular condyles.

The quadrate branches have a short longitudinal development compared to those of the lobes of the squamosals (Fig 9A and 9B). The dorsal surface of the quadrates is smooth except in the contact area with the quadratojugal, where it is slightly ornamented. The rear portion of the quadrates is flat, but rises slightly in the region of the otic recesses. The quadrates form the ventral margins of cranioquadrate grooves, which are posterolaterally open because the posterior absence of quadrate-squamosal contact (Fig 10C and 10D). The foramen aëreum is placed dorsally, close to the medial edge of the quadrates (Fig 9A and 9B). Both condyles have slightly elevated dorsal margins (Fig 10A and 10B). The lateral condyles are larger than the medial. A dorsal notch between them is observed. Moreover, the medial condyles are ventrally directed. In ventral view, a well-marked longitudinal ridge, extending parallel to the suture of the quadrates with the quadratojugals, is observed. In addition, a shorter second ridge extends parallel to the medial edges of the quadrates (Fig 9C and 9D).

The palatines are thick and elongate. They constitute most of the medial walls of the suborbital fenestrae (Fig 9C and 9D). They have parallel lateral edges in the posterior half, but the edges are slightly widened at the anterior half. Rostrally, the palatines form a wide and rounded process, which extends beyond the anterior margins of the suborbital fenestrae. The sutures of the palatines with the pterygoid have a zigzag pattern, and they reach the posteromedial region of the suborbital fenestrae.

The ectopterygoids are thick and form the posterolateral margins of the suborbital fenestrae (Fig 9C and 9D). Anteriorly, each projects a short process along the medial maxillary margin, forming the medial wall of the last two alveoli. The posterior processes of the ectopterygoids are long and robust. These bones show a linear medial suture with the pterygoids that does not reach the posterior margins of the pterygoid wings. The dorsal processes contact the ventral bases of the postorbital bars. The exact relationship of the ectopterygoids with the postorbitals is not known (Fig 10C and 10D).

The pterygoids are markedly expanded caudoventrally, and the posterolateral margins of the pterygoid wings almost reach the posterior edge of the quadrate (Fig 9C and 9D). Nevertheless, the pterygoid wings extend beyond the posterior margin of the posteromedial pterygoid processes, which are well-developed and posteriorly expanded. Despite deformation of the basioccipital region, the relative short pterygoids can be seen ventral to the median Eustachian opening. The choana is rounded, being posteroventrally projected. It is completely surrounded by the pterygoids and placed in a slightly depressed area in the middle of the pterygoid surface.

Although lacking well-preserved margins, the laterosphenoids seem to be wide and laterally expanded.

The supraoccipital is subtrapezoidal and wider than high in occipital view (Fig 10A and 10B). The postemporal fenestrae cannot be recognized due to the occipital region of the skull is collapsed. The supraoccipital has a marked sagittal crest beginning at the dorsal margin and extending longitudinally along the supraoccipital. Despite its poor preservation, a dorsal exposure of the supraoccipital is preserved on the posteromedial portion of the skull table (Fig 9A and 9B).

The exoccipitals constitute much of the occipital area of the skull and form the dorsolateral edge of the foramen magnum, both exoccipitals contacting above it. In its dorsal margin, the exoccipitals are very depressed (Fig 10A and 10B). The paroccipital processes extend laterally beyond the margin of the medial condyle of the quadrate. A thick tubercle on the ventral edge of the paroccipital process can be observed in other allodaposuchids; although Martin (2010) [22] stated that the tubercles are not present in the specimen from Fox-Amphoux, it is recognized in the right paroccipital process. Two foramina are distinguished in the medioventral area of the right exoccipital, near the occipital condyle. They may correspond to the hypoglossal (XII) and vagus foramina. In lateral view, the exoccipitals form the ventral half of the internal wall of the otic aperture, posteriorly preventing the contact between squamosal and quadrate (Fig 10C and 10D).

Deformation of the braincase displaced the medial processes of the pterygoids, which are hidden the basisphenoid. Due to this, we can neither provide an accurate description of this element in occipital view nor observe the basisphenoid rostrum.

The basioccipital is trapezoidal in occipital view, being wider than high (Fig 10A and 10B). Its dorsal half constitutes the occipital condyle, which lacks a well-developed neck. The basioccipital plate is vertical and bears a longitudinal sagittal crest. Deformation does not allow precise assessment of the ventral margin of the basioccipital.

#### Dentition

The dental formula of *Lohuecosuchus mechinorum* includes five premaxillary alveoli and twelve or thirteen maxillary alveoli. The two teeth preserved in the maxilla have conical morphology and are pointed and slightly curved ventrolingually. Their lingual surfaces are flat and their mesial and distal margins have lingually displaced longitudinal carinae. The labial surfaces are markedly convex, imparting a D-shape to the cross-section of the crown. The enamel is not ornamented.

## Results and Discussion

### Comparisons of *Lohuecosuchus* with other allodaposuchids

Within Allodaposuchidae, *Lohuecosuchus* is characterized by a wide and short rostrum. The other taxa included in this clade are also brevirostral, but with a slightly more elongated rostrum than that of *Lohuecosuchus*. Both *Lohuecosuchus megadontos* and *Lohuecosuchus mechinorum* possess large, laterally broad external nares that are wider than long. This kind of naris is not common within Eusuchia, and only some alligatoroids such as *Deinosuchus*, *Orthogenysuchus* or *Mourasuchus*, or the putative eusuchian *Pietraroiasuchus*, have similar nares. Within Allodaposuchidae, *Al*. *precedens* has a circular external naris and those of *Ar*. *gascabadiolorum* and *Al*. *subjuniperus* are oval-shaped.

The shallow, lateromedially expanded depressions on the dorsal maxillary surface of both species of *Lohuecosuchus* could be associated with the well-developed ridges that show the maxilla in dorsal view. Similar maxillary depressions appear in *Goniopholis*, although with a more posterior location, whereas the marked ridges are characteristic in *Boverisuchus*, *Mourasuchus* and some crocodylids. Other allodaposuchids do not share both characters.

*Lohuecosuchus* has a wide and rounded U-shaped anterior palatine process different from those of *Al*. *precedens* and *Al*. *subjuniperus*, whose anterior projection is short and scarcely pointed. However, this anterior process in *Lohuecosuchus megadontos* does not extend beyond the anterior margin of the suborbital fenestra, whereas in *Lohuecosuchus mechinorum* clearly exceeds these margins.

The pterygoid rami of the ectopterygoids are straight in *Lohuecosuchus*, constituting the linear posterolateral margins of the suborbital fenestra. This character is different in the other members of Allodaposuchidae. In this sense, *Al*. *precedens* and *Al*. *subjuniperus* have bowed pterygoid branches of the ectopterygoids that produce concave margins of the suborbital fenestra. However, the ectopterygoids are poorly preserved in *Al*. *precedens*. This feature is very variable within Eusuchia. In fact, some hylaeochampsids present linear posterolateral margins of the suborbital fenestra (i.e. *Hylaeochampsa* and *Iharkutosuchus*), whereas these margins are bowed in *Acynodon*. In Crocodylia the character also shows variability within its different lineages.

As in HUE-02920 and HUE-04263, paratypes of *L*. *megadontos*, each premaxilla in *L*. *mechinorum* bears five alveoli, with the fourth being the largest and the first and second the smallest. This character is shared with *Al*. *precedens*. However, it exists intraspecific variability within *L*. *megadontos*. In this way, the holotype bears four premaxillary alveoli, like *Al*. *subjuniperus*.

*Lohuecosuchus* shares with other Iberian allodaposuchids as *Al*. *subjuniperus* or *Ar*. *gascabadiolorum*, but not with the Romanian *Al*. *precedens*, the presence of a notch between the premaxilla and the maxilla, for the reception of the fourth mandibular tooth. This character is shown by all main lineages of Crocodylia except Alligatoroidea. This last clade shares the existence of a pit for the reception of the fourth mandibular tooth with *Al*. *precedens* and hylaeochampsids.

Another exclusive feature of the western European forms within Eusuchia is the presence of a small medial jugal foramen. Large medial jugal foramina are common in *Borealosuchus*, *Diplocynodon*, and crocodyloids, but most eusuchians have a smaller foramen in this area; *Al*. *precedens* is the only member of Allodaposuchidae with a large foramen.

The dental occlusion pattern is a further difference within Allodaposuchidae. Whereas the occlusion between dentary teeth and maxillary teeth in *Al*. *precedens* was considered tentatively as in-line by Delfino et al. (2008) [28], the Ibero-Armorican allodaposuchids present a lingual occlusion pattern.

This set of differences among allodaposuchids from Western Europe and *Al*. *precedens*, from Eastern Europe indicates a divergence within the clade above the species level, probably due to vicariance patterns caused by isolation of the eastern and western faunas, as has been observed in other vertebrate clades [46, 47].

Within *Lohuecosuchus*, *L*. *megadontos* has a broader snout than *L*. *mechinorum*. The latter has a rostral profile similar to the rest of allodaposuchids and is slightly elongate in its anterior region.

*Lohuecosuchus megadontos* specimens have the smallest number of maxillary alveoli within Allodaposuchidae. This reduced number of alveoli, ten or eleven, is related to the large size of the teeth, which makes the interalveolar spaces very small. *Lohuecosuchus mechinorum* bears twelve or thirteen alveoli, similar to *Al*. *precedens* (thirteen or fourteen) but fewer than *Ar*. *gascabadiolorum* (as many as fifteen) and *Al*. *subjuniperus* (fourteen). On the other hand, the teeth in *L*. *mechinorum* are similar in size to those of the representatives of the clade, and thus interalveolar spaces are not as narrow as in *L*. *megadontos*.

The peculiar morphology of the jugal and the ectopterygoid behind the tooth row observed in *L*. *megadontos* is also recognized as associated with the great dental development of this taxon. In this region, a marked jump or vaulting between the rear part of the maxillary branch and the anteroventral jugal process compressed ectopterygoid occur. This character is exclusive both within Allodaposuchidae and within Eusuchia.

A remarkable difference between *L*. *mechinorum* and other allodaposuchids is the large size of its fifth maxillary alveolus which is almost of the same size of the fourth. The presence of alveoli with the same size in fourth and fifth positions of the teeth row is common in several non-eusuchian neosuchians such as *Theriosuchus*, *Goniopholis*, *Bernissartia* and *Susisuchus*, and in crocodylians such as *Borealosuchus*, planocraniids, and basal members of Alligatoroidea and Crocodyloidea. However despite the large size of the fifth alveolus in *L*. *mechinorum*, the fourth is slightly larger, as in all the allodaposuchids.

The dorsal surface of the rostrum in *L*. *megadontos* bears ridges, bulges and depressions not present in *L*. *mechinorum* or in the other allodaposuchids. Behind the naris laterally, there are shallow depressions in the premaxillary surface of *L*. *megadontos*, as in several alligatoroids (i.e. *Procaimanoidea*, *Arambourgia*, *Alligator*). In addition, this species bears well-developed canthi rostralii similar to those of *Caiman* and *Melanosuchus*. Thus, the presence of both characters in *L*. *megadontos* is exclusive outside Crocodylia.

The incisive foramen is small and rounded in *L*. *megadontos*, whereas *L*. *mechinorum* has a small and elongated incisive foramen, probably due to the distortion, but similar to the poorly-preserved foramen in *Ar*. *gascabadiolorum*. The morphology of this foramen in *L*. *megadontos* is exclusive within Allodaposuchidae. A high variability in this character is known in this clade. Thus, the incisive foramen is almond-shaped in *Al*. *precedens* and teardrop-shaped in *Al*. *subjuniperus*. However, intraspecific variability cannot be excluded [8].

The parasagittal pair of foramina located in the middle of the medial edge of the palatal shelf in each maxilla of *L*. *megadontos* is not observed in any other allodaposuchid. A pair of foramina in this position is recognized, for example, in some species of *Goniopholis*, *Pelagosaurus* or *Notosuchus*, but not within Eusuchia.

*Lohuecosuchus megadontos* has a unique position of the jugal-quadratojugal suture among allodaposuchids. The suture forms the posterolateral angle of the infratemporal fenestra. Elsewhere in Eusuchia, this feature is only shared by *Deinosuchus* and some crocodyloids (i. e. *Tomistoma*, *Australosuchus* or *Kambara*).

The anterior process of the frontal of *L*. *megadontos*, extending beyond the anterior margin of the prefrontals, can also be observed in *Al*. *precedens* but not in other allodaposuchids.

The choana is posteroventrally oriented in all allodaposuchids, but its morphology varies within the clade. In this way, *L*. *megadontos* has an anteroposteriorly elongated, teardrop-shaped choana, *Lohuecosuchus mechinorum* bears a rounded choana, and *Ar*. *gascabadiolorum* seems to have an oval-shaped choana.

*Arenysuchus gascabadiolorum* was originally considered the oldest and most primitive crocodyloid of Europe (Puértolas et al., 2011, Puértolas-Pascual et al., 2013), and later a taxon closely related to *Allodaposuchus* (Blanco et al., 2014). The presence of a broad cranioquadrate passage laterally open and a shallow fossa in the anteromedial corner of the supratemporal fenestra justifies the inclusion of *Arenysuchus* within Allodaposuchidae. Despite this, *Arenysuchus* presents significant differences with *Lohuecosuchus*. The premaxilla of *Arenysuchus* is longer than wide, but it is difficult to know the precise number of alveoli. By contrast, in *Lohuecosuchus* the premaxilla is wider than long. In *Arenysuchus* the maxilla is slender, elongate and could bear fifteen alveoli, at least two to four more than in *Lohuecosuchus*. The unsculpted anterior frontal process of *Arenysuchus* might also extend beyond the anterior border of the prefrontals, whereas in *L*. *megadontos* this process is ornamented and does not exceed the anterior margin of the prefrontals. Both palatines and ectopterygoids are interpreted as slender and elongate elements in *Arenysuchus*, whereas the palatines are broad and the ectopterygoids thick in *Lohuecosuchus*.

Although not included in the phylogenetic analysis due to their fragmentary and partially problematic information, *Al*. *palustris*, *Mu*. *buffetauti* and *Ma*. *affuvelensis* can be recognized as different to *Lohuecosuchus*. The fragmentary specimen described as *Al*. *palustris* [6] allows a partial comparison with *Lohuecosuchus*. *Allodaposuchus palustris* is recognized as an allodaposuchid due to its upturned orbital margins, lack of contact between the quadrate and squamosal on the external surface of the skull posterior to the auditory aperture, lack of a defined posteroventral margin of the otic aperture, dermal bones overhanging the rim of the supratemporal fenestra, laterally open cranioquadrate passage represented by a sulcus, and dorsally placed quadrate foramen aëreum. In addition, *Al*. *palustris* presents distinct exclusive features such as the large quadrate foramen aëreum, the wide and short anterior frontal process, and teeth with well-marked ornamentation developing false-ziphodont crenulations.

However, some autapomorphies used by Blanco et al. (2014) [6] to define *Al*. *palustris* should be discussed. These authors argue that *Al*. *palustris* does not present a fossa at the anteromedial margin of the supratemporal fenestra. As commented, this shallow fossa in the anteromedial corner of the supratemporal fenestra is present in all representatives of Allodaposuchidae and absent in the other eusuchians. Interpretation of this character is problematic in *Al*. *palustris* due to the fragmentary nature of the material. The anterior wall of this fenestra in the holotype of *Al*. *palustris* is not vertical, and the presence of a shallow fossa at the rostromedial corner cannot be discounted. Regarding the frontoparietal suture, Blanco et al. (2014) [6] assume that it is concavo-convex. However, although this suture bears a slight curvature in its middle area, it may be considered as linear, as it does not have the more deeply curved morphology observed in groups like planocraniids, some gavialoids, *Leidyosuchus*, *Brachychampsa*, caimanines, some alligatorines and many crocodyloids. In these groups the contact between frontal and parietal describes a well-marked zigzag shaped suture, not as smooth as in *Al*. *palustris*. Therefore, this character state is shared with the other allodaposuchids. Blanco et al. (2014) [6] consider that the paraoccipital process of *Al*. *palustris* lacks the development of a boss in its ventrolateral margin, contrasting with the condition defined here for the allodaposuchids. This is a character closely related to the laterally opened cranioquadrate passage. Although the holotype and only known specimen of *Al*. *palustris* does not bear a boss as marked as in other allodaposuchid taxa, a thickening in the region of paraoccipital process, located medially to the cranioquadrate passage, is present. In fact, *Al*. *palustris* was considered by Martin et al. (2015) [8] as an invalid species due to the lack of diagnostic characters. In the same sense, there are no evident diagnostic characters in the recently proposed *Al*. *hulki*, and it should be taken with caution pending further review.

Although initially described as a basal alligatoroid [13, 15], the assignment of *Mu*. *buffetauti* to that clade was subsequently considered as problematic [3, 23, 29]. *Musturzabalsuchus* shares with the other allodaposuchids an exclusive character combination: tenth alveolus is the largest behind the fourth in the dentary tooth row; absence of an external mandibular fenestra; and the festooned dorsolateral surface of the dentary, forming two concave waves. The maxillae of both *Musturzabalsuchus* and *L*. *megadontos* possess closely spaced alveoli, with narrow interalveolar spaces. However, *Musturzabalsuchus* and *Lohuecosuchus* differ in characters of both the maxilla and the dentition. In *Musturzabalsuchus*, the maxilla is characterized by the presence of a well-marked festooning and ventrolaterally projected teeth in the anterior region, features not present in *Lohuecosuchus*. In addition, the conical teeth in *Musturzabalsuchus* are relatively much smaller than in *L*. *megadontos*. The number of maxillary alveoli is thirteen in *Musturzabalsuchus*, the same as the left maxillary branch in *L*. *mechinorum*, whereas no more than eleven are present in *L*. *megadontos*. In relation to the lower jaw, in *Mu*. *buffetauti* and *L*. *megadontos* the splenial does not participate in the symphysis, which extends to the fourth or fifth alveolus of the dentary, and the larger alveolus behind the fourth tooth is the tenth, a feature not shared with other basal eusuchians. Several features distinguish the lower jaws of *Mu*. *buffetauti* and *L*. *megadontos*. Although the general morphology of the teeth and crown surfaces are similar in the lower jaw of both taxa, with anterior pointed teeth and posterior blunt teeth, in *L*. *megadontos* the dentition is relatively larger. In addition, the lower jaw of *L*. *megadontos* is more robust than that of *Musturzabalsuchus*. *Lohuecosuchus megadontos* possesses a broad shelf formed by the surangular that it is not observed in *Musturzabalsuchus*.

Another Late Cretaceous crocodyliform first identified as a basal alligatoroid is *Ma*. *affuvelensis* [16]. The authors included *Ma*. *affuvelensis* as a member of Alligatoroidea based on several characters that they considered diagnostic of the clade. These were the lingual occlusion between the maxilla and mandible; the presence of a pit for the reception of the fourth mandibular tooth between the maxilla and the premaxilla; short and blunt crowns in the posteriormost maxillary teeth and lower jaw teeth; fourth tooth being the largest of the lower jaw teeth row. Lingual occlusion between the dentary and maxillary teeth is common in alligatoroids in contrast to the linear occlusion presented by gavialoids, and derived crocodyloids and species of *Borealosuchus*. However, lingual occlusion also appears in hylaeochampsids, planocraniids and all allodaposuchids. A pit for the reception of the fourth mandibular tooth is present in hylaeochampsids, alligatoroids (except *Leidyosuchus*) and some non-eusuchian neosuchians. This character is also present in *Al*. *precedens*. Nevertheless, neither the existence in *Massaliasuchus* of this pit nor the presence of a notch, as in the rest of allodaposuchids, is clear. The presence of short and blunt crowns of the posteriormost maxillary and lower jaw teeth is usual in alligatoroids, but also in *L*. *megadontos* and *Musturzabalsuchus*. In addition, Martin and Buffetaut (2008) [16] also suggested that *Massaliasuchus* could share with basal alligatoroids the presence of more elongate lacrimals than prefrontals and nasals without contact with the rear edge of the naris. The presence of longer lacrimals than prefrontals is common in basal alligatoroids (*Leidyosuchus*, Diplocynodontinae, basal globidonts), caimanines, and crocodyloids, but it is also present in all allodaposuchids. On the other hand, Martin and Buffetaut (2008) [16] indicated an interesting character that could be shared between *Massaliasuchus* and Diplocynodontinae: the absence of dorsal contact of the nasals with the posterior margin of the naris. This character is also present in most of gavialoids, *Borealosuchus*, and some caimanines and crocodyloids. Within Allodaposuchidae, the condition is different, and nasals contact the posterior margin of the naris.

Given the poor preservation of the cranial and mandibular remains assigned to *Massaliasuchus*, only limited comparisons with other allodaposuchids are possible. The cranial material of *Massaliasuchus* preserves several differences with *Lohuecosuchus*, such as the narrow and elongated snout.

However, in the lower jaw of *Massaliasuchus* described by Matheron (1869) [14], whose current whereabouts are not known, it is possible to distinguish certain characters that link *Massaliasuchus* with Allodaposuchidae. First, the dorsal surface of the dentary tooth row figured by Matheron (1869, pl. 1) [14] is very similar to that of *Musturzabalsuchus*, with a marked festooned profile describing two convex waves. Matheron (1869) [14] indicates the presence of fifteen mandibular alveoli, a number similar to that of *Mu*. *buffetauti* (sixteen) and *Lohuecosuchus megadontos* (fourteen). The morphology of the tooth series is similar between this lower jaw, *Musturzabalsuchus* and *L*. *megadontos*, with pointed teeth in the anterior region and blunt teeth in the posterior region (character also present in alligatoroids). The lower jaw figured by Matheron (1869, pl. 1) [14], *Musturzabalsuchus* and *L*. *megadontos* also share the absence of external mandibular fenestra (a rare condition within Crocodylia, but also shared with hylaeochampsids and non-eusuchian neosuchians such as *Bernissartia*, *Shamosuchus*, *Rugosuchus*, *Pietraroiasuchus* or *Pachycheilosuchus*). Finally, the lower jaw figured by Matheron [14] could preserve one of the synapomorphies of Allodaposuchidae: the tenth alveolus being the largest behind the fourth in the dentary tooth row. Although the fifth, sixth and seventh teeth are not known in the drawing of Matheron (1869, pl. 1) [14], they are highly unlikely to be larger than the tenth. Taking into account all these characters, and the geographical and temporal distribution of *Ma*. *affuvelensis* (early Campanian) congruent with that of other allodaposuchids (late Campanian-early Maastrichtian), but not with European alligatoroids (all of which are of Cenozoic age), *Massaliasuchus* can be considered as member of Allodaposuchidae.

### Phylogenetic analysis

#### Method

A cladistic analysis has been performed in order to establish the phylogenetic relationships of *Lohuecosuchus* based on the data matrix of Brochu and Storrs (2012) [48]. Character 51 (largest dentary alveolus immediately caudal to fourth) has been modified because the lower jaw of *Lohuecosuchus* could not be codified with the existing states in the matrix. A new state, “the largest dentary alveolus immediately caudal to fourth be the tenth”, has been added. According to Delfino et al. (2008) [28], a new state has been added for the characters 148 (quadrate and squamosal not in contact on the external surface of the skull, posteriorly to the external auditory meatus) and 149 (caudal margin of otic aperture not defined and gradually merging into the exoccipital) due to the presence of a cranioquadrate passage laterally open in *Hylaeochampsa* and allodaposuchids.

Additionally, *Shamosuchus djadochtaensis* [49] (Campanian, Mongolia), *Pietraroiasuchus ormezzanoi* [11] (Albian, Italy), *Pachycheilosuchus trinquei* [50] (Albian, United States) and Late Cretaceous taxa *Al*. *subjuniperus*, *Ar*. *gascabadiolorum*, the type series of *Lohuecosuchus megadontos* and the holotype of *L*. *mechinorum* have been incorporated to the phylogenetic analysis, based on direct examination of fossil specimens. For *Al*. *precedens*, the re-scoring published by Blanco et al. (2014) [6] based on the specimen from Oarda de Jos (Romania) [28] has been used, with some modifications. Character 89 (position of incisive foramen) is recoded as 0, due to it does not reach the premaxillary teeth; character 105 (end of maxilla in palatal view with respect to infratemporal fenestra) is recoded as 0, because it terminates anterior to the lower temporal bar; character 117 (lateral edges of palatines) is recoded as 0, due to they are anteriorly smooth; character 119 (posterolateral margin of the suborbital fenestra) is recoded as 1, considering that it is concave; character 120 (lateral edges of palatines) is recoded as 0, due to they are parallel; character 142 (posterior angle of the infratemporal fenestra) is recoded as 0, because it is formed by the quadratojugal; character 152 (dermal bones of skull roof) is recoded as 1, due to the overhang the rim of the supratemporal fenestra; and character 181 (morphology and orientation of medial quadrate hemicondyle) is recoded as 0, because the hemicondyle is small and ventrally oriented. Character 131 (morphology of the anterior frontal process) has been considered as unknown, because the limits of the frontal are unclear in the only specimen of this taxon where this region is preserved. The final data matrix includes 103 taxa and 189 morphological characters. *Bernissartia fagesii* was set as outgroup. The matrix was analysed with TNT [51] using heuristic searches based on 1000 random addition sequence replicates. Bootstrap frequencies and Bremer support values have also been calculated using TNT.

#### Results

**Fig 11:**
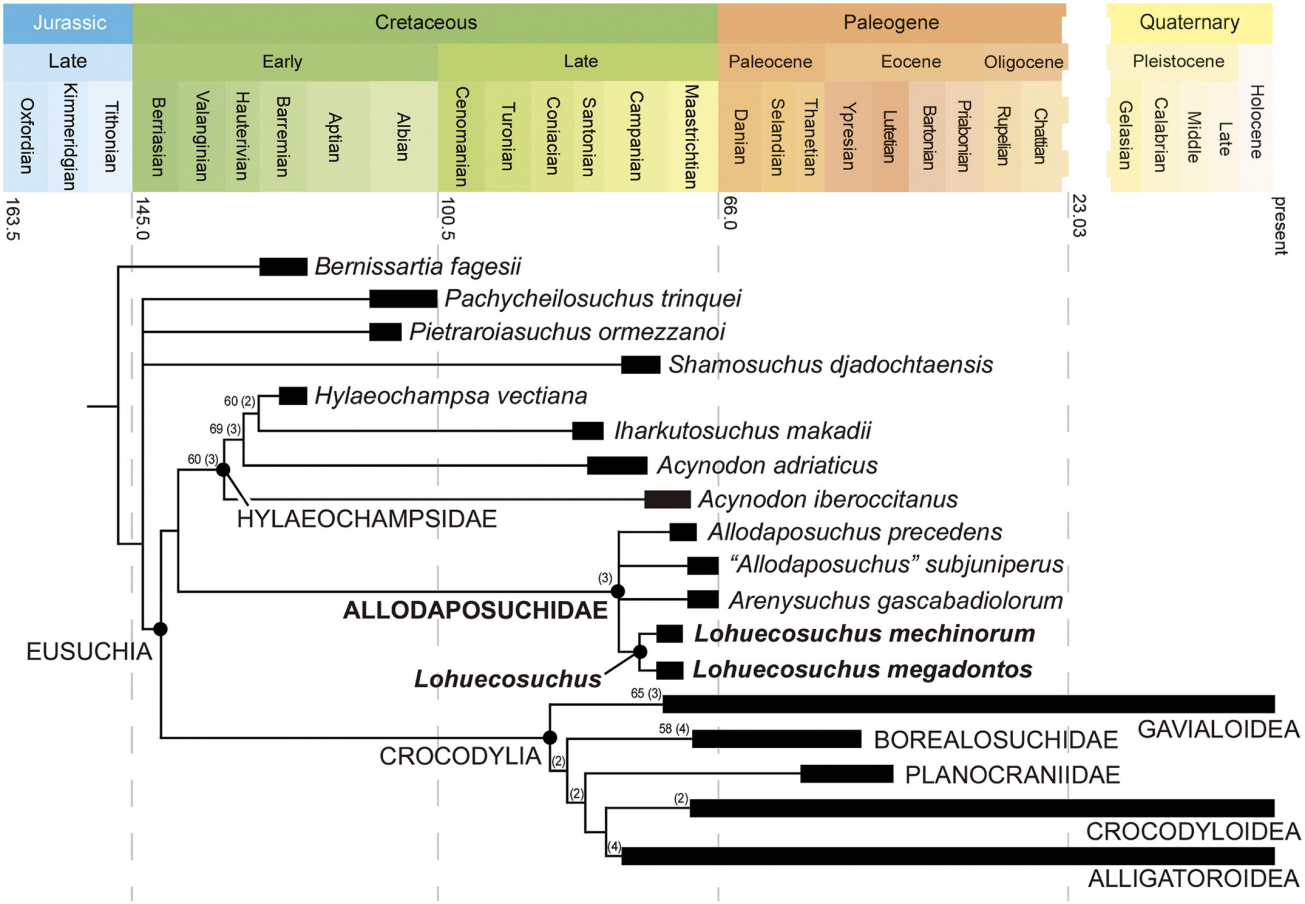
Calibrated cladogram corresponding to the strict consensus tree obtained in the phylogenetic analysis. The black rectangles represent the stratigraphic distribution of each taxon. Numbers at nodes indicate bootstrap frequencies over 50%, and Bremer support, over 2 (in parentheses).

The analysis recovered 8431 most parsimonious trees of 734 evolutionary steps, with a consistency index (CI) of 0,332 and a retention index (RI) of 0,798. The obtained topology (Fig 11) is consistent with those presented in some previous phylogenetic analyses [5, 9, 11, 12, 28], and monophyly of the higher groups of Eusuchia is recovered. The strict consensus reveals a monophyletic clade formed by Allodaposuchidae and Hylaeochampsidae, exclusively composed by European Cretaceous forms. This clade is recognized as the sister group of Crocodylia. *Pietraroiasuchus*, *Pachycheilosuchus* and the paralligatorid *Shamosuchus* are placed as non-eusuchian neosuchians, in contrast with the eusuchian affinities for these taxa suggested in several phylogenetic proposals [11, 27]. Therefore, Eusuchia is recognized here as composed by a monophyletic group formed by Hylaeochampsidae and Allodaposuchidae on the one hand, and Crocodylia on the other. In this analysis, Eusuchia is supported by the presence of dorsal osteoderms lacking keels (character 38, state 0), an ectopterygoid that abuts with the maxillary tooth row (character 104, state 0), a choana entirely surrounded by the pterygoids (character 121, state 1), and a posteriorly oriented external surface of the basioccipital ventral to the occipital condyle (character 170, state 1).

The European clade formed by hylaeochampsids and allodaposuchids is supported by the absence of anterior perforation for mandibular ramus of cranial nerve V (character 52, state 1); presence of a splenial excluded from mandibular symphysis, with its anterior tip ventral to Meckelian groove (character 54, state 1); external naris not bisected but contacted by nasals (character 82, state 1); pterygoid ramus of the ectopterygoid bowed, showing a concave posterolateral margin of suborbital fenestra (character 119, state 1); absence of contact of quadrate and squamosal on the external surface of the skull, posteriorly to the external meatus (character 148, state 0); and caudal margin of otic aperture not defined and gradually merging into the exoccipital (character 149, state 0).

Hylaeochampsidae is supported by the presence of procumbent anterior dentary teeth (character 48, state 0); a maxilla that comprises part of the lower temporal bar (character 105, state 1); the anterior tip of frontal forming broad and complex sutural contact with the nasals (character 131, state 1); a massive postorbital bar (character 133, state 0); reduced or absent quadratojugal spine (character 140, state 1); presence of a prominent knob on ventral surface of quadrate ramus (character 180, state 1); and an ectopterygoid maxillary ramus constituting more than two-thirds of the lateral margin of the suborbital fenestra (character 185, state 1).

Allodaposuchidae is supported by the presence of a maxillary posterior process within the lacrimal (character 128, state 1), ventral margin of the postorbital bar being part of the lateral jugal surface (character 135, state 1), upturned dorsal edges of the orbits (character 137, state 1), dermal bones of the skull roof overhanging the rim of the supratemporal fenestra (character 152, state 1), a shallow fossa at the anteromedial corner of the supratemporal fenestra (character 153, state 0), and a planar skull table surface (character 156, state 1).

Within Allodaposuchidae, *Al*. *precedens* is diagnosed here by the presence of an occlusion pit between the maxilla and the premaxilla (character 91, state 1); linear occlusion of the dentary teeth with the maxillary tooth row (character 92, state 2); very large medial jugal foramen (character 102, state 1); capitate process of laterosphenoid laterally oriented toward midline (character 166, state 0); anteroposteriorly wide basisphenoid ventral to the basioccipital (character 172, state 1); and basisphenoid exposed as a broad sheet ventral to the basioccipital (character 173, state 0). *Allodaposuchus subjuniperus* is supported by the dorsal projection of the naris (character 81, state 1), presence of four teeth in the premaxilla (character 87, state 1), and massive postorbital bar (character 133, state 0). *Arenysuchus gascabadiolorum* does not present any synapomorphy in this analysis.

The genus *Lohuecosuchus* is supported by a naris wider than long (character 83, state 1), presence of prominent preorbital ridges (character 97, state 1), and straight pterygoid ramus of the ectopterygoid, resulting in a linear posterolateral margin of the suborbital fenestra (character 119, state 0). *Lohuecosuchus mechinorum* is supported by the presence of a massive postorbital bar (character 133, state 0), and basisphenoid not broadly exposed ventral to the basioccipital, the pterygoid being short ventral to the median Eustachian opening (character 173, state 1). *Lohuecosuchus megadontos* is supported by the presence of deep notch lateral to the naris (character 86, state 1), prominent canthi rostralii (character 96, state 1), and quadratojugal-jugal suture forming the posterior angle of infratemporal fenestra (character 142, state 2).

### Phylogenetic and paleobiogeographical implications

Allodaposuchidae, as comprised of *Allodaposuchus precedens* and crocodyliforms closer to it than to hylaeochampsids, paralligatorids, or crocodylians, has several phylogenetic as paleobiogeographical implications. First, Allodaposuchidae and Hylaeochampsidae are identified as two branches of a clade recognized as the sister group of Crocodylia, the only lineage of Crocodyliformes that persists to the present. It is remarkable that both Allodaposuchidae as Hylaeochampsidae, as currently known, include exclusively European Cretaceous forms.

The conformation of Europe during the Late Cretaceous, as an archipelago, favored vicariance and endemism with groups of terrestrial vertebrates such as dinosaurs, squamates, turtles and crocodyliforms [46, 47]. In this sense, examples of these distribution patterns, both at generic level and at higher taxonomic ranges, have been recognized in several European Late Cretaceous clades [46, 47, 52, 53]. Allodaposuchidae is recognized as an endemic group of the European archipelago, with a limited known temporal range, from the early Campanian to the late Maastrichtian. This clade is known in two areas: it is represented by an endemic form from the Transylvanian region, *Al*. *precedens*, only known in the early Maastrichtian; and it is recognized by several early Campanian to late Maastrichtian Ibero-Armorican forms (*Al*. *subjuniperus*, *Al*. *palustris*, *Al*. *hulki*, *Ar*. *gascabadiolorum*, *Mu*. *buffetauti* and *L*. *megadontos* from Spain; and *L*. *mechinorum* from France), showing a set of characters not present in the Transylvanian specimens (e.g. presence of a notch between maxilla and premaxilla for the reception of the fourth mandibular teeth or linear occlusion between maxillary teeth and mandibular teeth).

Differences among the Ibero-Armorican allodaposuchids and *Al*. *precedens* can be explained by a model of vicariance. The distribution pattern of Allodaposuchidae is similar to that proposed by Weishampel et al. (2010) [54] for other clades, suggesting that the Romanian faunas were very similar to those from other European regions, but geographical isolation of the islands during the Late Cretaceous favored the emergence of endemic taxa. Therefore, we propose restriction of the name *Allodaposuchus* to specimens from Eastern Europe.

Inclusion of *Ar*. *gascabadiolorum*, *Mu*. *buffetauti* and *Ma*. *affuvelensis* within Allodaposuchidae allows us to refute the presence of both Alligatoroidea and Crocodyloidea in European Late Cretaceous record. *Arenysuchus* appears to clearly be an allodaposuchid, and although information on *Musturzabalsuchus* and *Massaliasuchus* is scarce, there is nothing in their morphology that uniquely argues for an alligatoroid affinity and they, too, share diagnostic characters with allodaposuchids. Assuming thoracosaurs are gavialoids [18], the only crocodylian lineage known from the Mesozoic of Europe appears to be Gavialoidea [55, 56].

Therefore, the eusuchians found in the Late Cretaceous of Eurasia are recognized here as belonging to two closely related clades endemic to Europe: Allodaposuchidae and Hylaeochampsidae. The disappearance of allodaposuchids and hylaeochampsids in Europe, as well as paralligatorids in Asia [27, 57], may have allowed their close relatives, the crocodylians, to prosper throughout the Northern Hemisphere after the Cretaceous-Paleogene mass extinction.

As can be observed in this study, some states of character defining the node Allodaposuchidae in the present phylogenetic analysis are shared with several representatives of Crocodylia. Thus, the presence of some character states in basal eusuchians that previously diagnosed lineages as Alligatoroidea or Crocodyloidea may suggest plesiomorphy at broader phylogenetic levels.

## Supporting Information

S1 Supporting Information List of characters from Brochu and Storrs (2012) and modifications carried out on some characters used in the phylogenetic analysis. (PDF)

S2 Supporting Information Data Matrix. (TNT)

S3 Supporting Information List of several measurements of the skull of the holotypes of the two known species of *Lohuecosuchus*, HUE-04498 (*L*. *megadontos*) and MDE/CM-616 (*L*. *mechinorum*). (PDF)
