# Supplementary material for: New Crocodyliforms from Southwestern Europe and Definition of a Diverse Clade of European Late Cretaceous Basal Eusuchians
Source: PLoS One. 2015 Nov 4;10(11):e0140679. doi: 10.1371/journal.pone.0140679 (PMC4633049; doi:10.1371/journal.pone.0140679)
Supplement: S1 Supporting Information — (PDF) [file pone.0140679.s001.pdf]

## S1. List of characters from Brochu and Storrs (2012) and modifications carried out on some characters used in the phylogenetic analysis.

(1) Ventral tubercle of proatlas more than one-half (0) or no more than one-half (1) the width of the dorsal crest.

(2) Fused proatlas boomerang-shaped (0), strap-shaped (1), or massive and block-shaped (2).

(3) Proatlas with prominent anterior process (0) or lacks anterior process (1).

(4) Proatlas has tall dorsal keel (0) or lacks tall dorsal keel; dorsal side smooth (1).

(5) Atlas intercentrum wedge-shaped in lateral view, with insignificant parapophyseal processes (0), or plate-shaped in lateral view, with prominent parapophyseal processes at maturity (1).

(6) Dorsal margin of atlantal rib generally smooth with modest dorsal process (0) or with prominent process (1).

(7) Atlantal ribs without (0) or with (1) very thin medial laminae at anterior end.

(8) Atlantal ribs lack (0) or possess (1) large articular facets at anterior ends for each other.

(9) Axial rib tuberculum wide, with broad dorsal tip (0) or narrow, with acute dorsal tip (1).

(10) Axial rib tuberculum contacts diapophysis late in ontogeny, if at all (0) or early in ontogeny (1).

(11) Anterior half of axis neural spine oriented horizontally (0) or slopes anteriorly (1).

(12) Axis neural spine crested (0) or not crested (1).

(13) Posterior half of axis neural spine wide (0) or narrow (1).

(14) Axis neural arch lacks (0) or possesses (1) a lateral process (diapophysis).

(15) Axial hypapophysis located toward the center of centrum (0) or toward the anterior end of centrum (1).

(16) Axial hypapophysis without (0) or with (1) deep fork.

(17) Hypapophyseal keels present on eleventh vertebra behind atlas (0), twelfth vertebra behind atlas (1), or tenth vertebra behind atlas (2).

(18) Third cervical vertebra (first postaxial) with prominent hypapophysis (0) or lacks prominent hypapophysis (1).

(19) Neural spine on third cervical long, dorsal tip at least half the length of the centrum without the cotyle (0) or short, dorsal tip acute and less than half the length of the centrum without the cotyle (1).

(20) Cervical and anterior dorsal centra lack (0) or bear (1) deep pits on the ventral surface of the centrum.

(21) Presacral centra amphicoelous (0) or procoelous (1).

(22) Anterior sacral rib capitulum projects far anteriorly of tuberculum and is broadly visible in dorsal view (0), or anterior margins of tuberculum and capitulum nearly in same plane, and capitulum largely obscured dorsally (1).

(23) Scapular blade flares dorsally at maturity (0) or sides of scapular blade subparallel; minimal dorsal flare at maturity (1).

(24) Deltoid crest of scapula very thin at maturity, with sharp margin (0) or very wide at maturity, with broad margin (1).

(25) Scapulocoracoid synchondrosis closes very late in ontogeny (0) or relatively early in ontogeny (1).

(26) Scapulocoracoid facet anterior to glenoid fossa uniformly narrow (0) or broad immediately anterior to glenoid fossa, and tapering anteriorly (1).

(27) Proximal edge of deltopectoral crest emerges smoothly from proximal end of humerus and is not obviously concave (0) or emerges abruptly from proximal end of humerus and is obviously concave (1).

(28) M. teres major and M. dorsalis scapulae insert separately on humerus; scars can be distinguished dorsal to deltopectoral crest (0) or insert with common tendon; single insertion scar (1).

(29) Olecranon process of ulna narrow and sub-angular (0) or wide and rounded (1).

(30) Distal extremity of ulna expanded transversely with respect to long axis of bone; maximum width equivalent to that of proximal extremity (0) or proximal extremity considerably wider than distal extremity (1).

(31) Interclavicle flat along length, without dorsoventral flexure (0) or with moderate dorsoventral flexure (1) or with severe dorsoventral flexure (2).

(32) Anterior end of interclavicle flat (0) or rod-like (1).

(33) Iliac anterior process prominent (0) or virtually absent (1).

(34) Dorsal margin of iliac blade rounded with smooth border (0) or rounded, with modest dorsal indentation (1) or rounded, with strong dorsal indentation (wasp-waisted; 2) or narrow, with dorsal indentation (3) or rounded with smooth border; posterior tip of blade very deep (4).

(35) Supraacetabular crest narrow (0) or broad (1).

(36) Limb bones relatively robust, and hind limb much longer than forelimb at maturity (0) or limb bones very long and slender (1).

(37) M. caudofemoralis with single head (0) or with double head (1).

(38) Dorsal osteoderms not keeled (0) or keeled (1).

(39) Dorsal midline osteoderms rectangular (0) or nearly square (1).

(40) Four (0), six (1), eight (2), or ten (3) contiguous dorsal osteoderms per row at maturity.

(41) Nuchal shield grades continuously into dorsal shield (0) or differentiated from dorsal shield; four nuchal osteoderms (1) or differentiated from dorsal shield; six nuchal osteoderms with four central and two lateral (2) or differentiated from dorsal shield; eight nuchal osteoderms in two parallel rows (3).

(42) Ventral armor absent (0) or single ventral osteoderms (1) or paired ventral ossifications that suture together (2).

(43) [40] Anterior margin of dorsal midline osteoderms with anterior process (0) or smooth, without process (1).

(44) Ventral scales have (0) or lack (1) follicle gland pores.

(45) Ventral collar scales not enlarged relative to other ventral scales (0) or in a single enlarged row (1) or in two parallel enlarged rows (2).

(46) Median pelvic keel scales form two parallel rows along most of tail length (0) or form single row along tail (1) or merge with lateral keel scales (2).

(47) Alveoli for dentary teeth 3 and 4 nearly same size and confluent (0) or fourth alveolus larger than third, and alveoli are separated (1).

(48) Anterior dentary teeth strongly procumbent (0) or project anterodorsally (1).

(49) Dentary symphysis extends to fourth or fifth alveolus (0) or sixth through eighth alveolus (1) or behind eighth alveolus (2).

(50) Dentary gently curved (0), deeply curved (1), or linear (2) between fourth and tenth alveoli.

(51) Largest dentary alveolus immediately caudal to fourth is (0) 13 or 14, (1) 13 or 14 and a series behind it, (2) 11 or 12, (3) no differentiation, (4) behind 14, (5) 10. (Modified from Brochu & Storrs, 2012).

(52) Splenial with anterior perforation for mandibular ramus of cranial nerve V (0) or lacks anterior perforation for mandibular ramus of cranial nerve V (1).

(53) Mandibular ramus of cranial nerve V exits splenial anteriorly only (0) or splenial has singular perforation for mandibular ramus of cranial nerve V posteriorly (1) or splenial has double perforation for mandibular ramus of cranial nerve V posteriorly (2).

(54) Splenial participates in mandibular symphysis; splenial symphysis adjacent to no more than five dentary alveoli (0) or splenial excluded from mandibular symphysis; anterior tip of splenial passes ventral to Meckelian groove (1) or splenial excluded from mandibular symphysis; anterior tip of splenial passes dorsal to Meckelian groove (2) or deep splenial symphysis, longer than five dentary alveoli; splenial forms wide ‘V’ within symphysis (3) or deep splenial symphysis, longer than five dentary alveoli; splenial constricted within symphysis and forms narrow ‘V’ (4).

(55) Coronoid bounds posterior half of foramen intermandibularis medius (0) or completely surrounds foramen intermandibularis medius at maturity (1) or obliterates foramen intermandibularis medius at maturity (2).

(56) Superior edge of coronoid slopes strongly anteriorly (0) or almost horizontal (1).

(57) Inferior process of coronoid laps strongly over inner surface of Meckelian fossa (0) or remains largely on medial surface of mandible (1).

(58) Coronoid imperforate (0) or with perforation posterior to foramen intermandibularis medius (1).

(59) Process of splenial separates angular and coronoid (0) or no splenial process between angular and coronoid (1).

(60) Angular-surangular suture contacts external mandibular fenestra at posterior angle at maturity (0) or passes broadly along ventral margin of external mandibular fenestra late in ontogeny (1).

(61) Anterior processes of surangular unequal (0) or sub-equal to equal (1).

(62) Surangular with spur bordering the dentary toothrow lingually for at least one alveolus length (0) or lacking such spur (1).

(63) External mandibular fenestra absent (0) or present as narrow slit, no discrete fenestral concavity on angular dorsal margin (1) or present with discrete concavity on angular

dorsal argin (2) or present and very large; most of foramen intermandibularis caudalis visible in lateral view (3).

(64) Surangular-dentary suture intersects external mandibular fenestra anterior to posterodorsal corner (0) or at posterodorsal corner (1).

(65) Angular extends dorsally toward or beyond anterior end of foramen intermandibularis caudalis; anterior tip acute (0) or does not extend dorsally beyond anterior end of foramen intermandibularis caudalis; anterior tip very blunt (1).

(66) Surangular-angular suture lingually meets articular at ventral tip (0) or dorsal to tip (1).

(67) Surangular continues to dorsal tip of lateral wall of glenoid fossa (0) or truncated and not continuing dorsally (1).

(68) Articular-surangular suture simple (0) or articular bears anterior lamina dorsal to lingual foramen (1) or articular bears anterior lamina ventral to lingual foramen (2) or bears laminae above and below foramen (3).

(69) Lingual foramen for articular artery and alveolar nerve perforates surangular entirely (0) or perforates surangular/angular suture (1).

(70) Foramen aerum at extreme lingual margin of retroarticular process (0) or set in from margin of retroarticular process (1).

(71) Retroarticular process projects posteriorly (0) or projects posterodorsally (1).

(72) Surangular extends to posterior end of retroarticular process (0) or pinched off anterior to tip of retroarticular process (1).

(73) Surangular-articular suture oriented anteroposteriorly (0) or bowed strongly laterally (1) within glenoid fossa.

(74) Sulcus between articular and surangular (0) or articular flush against surangular (1).

(75) Dorsal projection of hyoid cornu flat (0) or rod-like (1).

(76) Dorsal projection of hyoid cornu narrow, with parallel sides (0) or flared (1).

(77) Lingual osmoregulatory pores small (0) or large (1).

(78) Tongue with (0) or without (1) keratinized surface.

(79) Teeth and alveoli of maxilla and/or dentary circular in cross-section (0), or posterior teeth laterally compressed (1), or all teeth compressed (2).

(80) Maxillary and dentary teeth with smooth carinae (0) or serrated (1).

(81) Naris projects anterodorsally (0) or dorsally (1).

(82) External naris bisected by nasals (0) or nasals contact external naris, but do not bisect it (1) or nasals excluded, at least externally, from naris; nasals and premaxillae still in contact (2) or nasals and premaxillae not in contact (3).

(83) Naris circular or keyhole-shaped (0) or wider than long (1) or anteroposteriorly long and prominently teardrop-shaped (2).

(84) External naris of reproductively mature males (0) remains similar to that of females or (1) develops bony excrescence (ghara).

(85) External naris (0) opens flush with dorsal surface of premaxillae or (1) circumscribed by thin crest.

(86) Premaxillary surface lateral to naris smooth (0) or with deep notch lateral to naris (1).

(87) Premaxilla has five teeth (0) or four teeth (1) early in posthatching ontogeny.

(88) Incisive foramen small, less than half the greatest width of premaxillae (0) or large, more than half the greatest width of premaxillae (1) or large, and intersects premaxillary-maxillary suture (2).

(89) Incisive foramen completely situated far from premaxillary tooth row, at the level of the second or third alveolus (0) or abuts premaxillary tooth row (1) or projects between first premaxillary teeth (2).

(90) Dorsal premaxillary processes short, not extending beyond third maxillary alveolus (0) or long, extending beyond third maxillary alveolus (1).

(91) Dentary tooth 4 occludes in notch between premaxilla and maxilla early in ontogeny (0) or occludes in a pit between premaxilla and maxilla; no notch early in ontogeny (1).

(92) All dentary teeth occlude lingual to maxillary teeth (0) or occlusion pit between seventh and eighth maxillary teeth; all other dentary teeth occlude lingually (1) or dentary teeth occlude in line with maxillary toothrow (2).

(93) Largest maxillary alveolus is 3 (0), 5 (1), 4 (2), 4 and 5 are same size (3), 6 (4), or maxillary teeth homodont (5), or maxillary alveoli gradually increase in diameter posteriorly toward penultimate alveolus (6).

(94) Maxillary tooth row curved medially or linear (0) or curves laterally broadly (1) posterior to first six maxillary alveoli.

(95) Dorsal surface of rostrum curves smoothly (0) or bears medial dorsal boss (1).

(96) Canthi rostralii absent or very modest (0) or very prominent (1) at maturity.

(97) Preorbital ridges absent or very modest (0) or very prominent (1) at maturity.

(98) Antorbital fenestra present (0) or absent (1).

(99) Vomer entirely obscured by premaxilla and maxilla (0) or exposed on palate at premaxillary-maxillary suture (1).

(100) Vomer entirely obscured by maxillae and palatines (0) or exposed on palate between palatines (1).

(101) Surface of maxilla within narial canal imperforate (0) or with a linear array of pits (1).

(102) Medial jugal foramen small (0) or very large (1).

(103) Maxillary foramen for palatine ramus of cranial nerve V small or not present (0) or very large (1).

(104) Ectopterygoid abuts maxillary tooth row (0) or maxilla broadly separates ectopterygoid from maxillary tooth row (1).

(105) Maxilla terminates in palatal view anterior to lower temporal bar (0) or comprises part of the lower temporal bar (1).

(106) Penultimate maxillary alveolus less than (0) or more than (1) twice the diameter of the last maxillary alveolus.

(107) Prefrontal dorsal surface smooth adjacent to orbital rim (0) or bearing discrete knob-like processes (1).

(108) Dorsal half of prefrontal pillar narrow (0) or expanded anteroposteriorly (1).

(109) Medial process of prefrontal pillar expanded dorsoventrally (0) or anteroposteriorly (1).

(110) Prefrontal pillar solid (0) or with large pneumatic recess (1).

(111) Medial process of prefrontal pillar wide (0) or constricted (1) at base.

(112) Maxilla has linear medial margin adjacent to suborbital fenestra (0) or bears broad shelf extending into fenestra, making lateral margin concave (1).

(113) Anterior face of palatine process rounded or pointed anteriorly (0) or notched anteriorly (1).

(114) Anterior ectopterygoid process tapers to a point (0) or forked (1).

(115) Palatine process extends (0) or does not extend (1) significantly beyond anterior end of suborbital fenestra.

(116) Palatine process generally broad anteriorly (0) or in form of thin wedge (1).

(117) Lateral edges of palatines smooth anteriorly (0) or with lateral process projecting from palatines into suborbital fenestrae (1).

(118) Palatine-pterygoid suture nearly at (0) or far from (1) posterior angle of suborbital fenestra.

(119) Pterygoid ramus of ectopterygoid straight, posterolateral margin of suborbital fenestra linear (0) or ramus bowed, posterolateral margin of fenestra concave (1).

(120) Lateral edges of palatines parallel posteriorly (0) or flare posteriorly, producing shelf (1).

(121) Anterior border of the choana is comprised of the palatines (0) or choana entirely surrounded by pterygoids (1).

(122) Choana projects posteroventrally (0) or anteroventrally (1) at maturity.

(123) Pterygoid surface lateral and anterior to internal choana flush with choanal margin (0) or pushed inward anterolateral to choanal aperture (1) or pushed inward around choana to form neck surrounding aperture (2) or everted from flat surface to form neck surrounding aperture (3).

(124) Posterior rim of internal choana not deeply notched (0) or deeply notched (1).

(125) Internal choana not septate (0) or with septum that remains recessed within choana (1) or with septum that projects out of choana (2).

(126) Ectopterygoid-pterygoid flexure disappears during ontogeny (0) or remains throughout ontogeny (1).

(127) Ectopterygoid extends (0) or does not extend (1) to posterior tip of lateral pterygoid flange at maturity.

(128) Lacrimal makes broad contact with nasal; no posterior process of maxilla (0) or maxilla with posterior process within lacrimal (1) or maxilla with posterior process between lacrimal and prefrontal (2).

(129) Prefrontals separated by frontals and nasals (0) or prefrontals meet medially (1).

(130) Lacrimal longer than prefrontal (0), or prefrontal longer than lacrimal (1), or lacrimal and prefrontal both elongate and nearly the same length (2).

(131) Anterior tip of frontal (0) forms simple acute point or (1) forms broad, complex sutural contact with the nasals.

(132) Ectopterygoid extends along medial face of postorbital bar (0) or stops abruptly ventral to postorbital bar (1).

(133) Postorbital bar massive (0) or slender (1).

(134) Postorbital bar bears process that is prominent, dorsoventrally broad, and divisible into two spines (0) or bears process that is short and generally not prominent (1).

(135) Ventral margin of postorbital bar flush with lateral jugal surface (0) or inset from lateral jugal surface (1).

(136) Postorbital bar continuous with anterolateral edge of skull table (0) or inset (1).

(137) Margin of orbit flush with skull surface (0) or dorsal edges of orbits upturned (1) or orbital margin telescoped (2).

(138) Ventral margin of orbit circular (0) or with prominent notch (1).

(139) Palpebral forms from single ossification (0) or from multiple ossifications (1).

(140) Quadratojugal spine prominent at maturity (0) or greatly reduced or absent at maturity (1).

(141) Quadratojugal spine low, near posterior angle of infratemporal fenestra (0) or high, between posterior and superior angles of infratemporal fenestra (1).

(142) Quadratojugal forms posterior angle of infratemporal fenestra (0) or jugal forms posterior angle of infratemporal fenestra (1) or quadratojugal-jugal suture lies at posterior angle of infratemporal fenestra (2).

(143) Postorbital neither contacts quadrate nor quadratojugal medially (0) or contacts quadratojugal, but not quadrate, medially (1) or contacts quadrate and quadratojugal at dorsal angle of infratemporal fenestra (2) or contacts quadratojugal with significant descending process (3).

(144) Quadratojugal bears long anterior process along lower temporal bar (0) or bears modest process, or none at all, along lower temporal bar (1).

(145) Quadratojugal extends to superior angle of infratemporal fenestra (0) or does not extend to superior angle of infratemporal fenestra; quadrate participates in fenestra (1).

(146) Postorbital-squamosal suture oriented ventrally (0) or passes medially (1) ventral to skull table.

(147) Dorsal and ventral rims of squamosal groove for external ear valve musculature parallel (0) or squamosal groove flares anteriorly (1).

(148) Quadrate and squamosal not in contact on the external surface of the skull, posteriorly to the external auditory meatus (0) or quadratosquamosal suture extends dorsally along caudal margin of the external auditory meatus (1) or extends only to the caudoventral corner of the external auditory meatus (2). (Modified by Delfino et al., 2008b from Brochu, 1999, and Salisbury et al., 2006, character 132).

(149) Caudal margin of otic aperture not defined and gradually merging into the exoccipital (0) or smooth and continuous with the paraoccipital process (1) or caudal margin of otic aperture inset (2). (Modified by Delfino et al., 2008b from Salisbury et al., 2006, character 102).

(150) Frontoparietal suture deeply within supratemporal fenestra; frontal prevents broad contact between postorbital and parietal (0) or suture makes modest entry into supratemporal fenestra at maturity; postorbital and parietal in broad contact (1) or suture on skull table entirely (2).

(151) Frontoparietal suture concavoconvex (0) or linear (1) between supratemporal fenestrae.

(152) Supratemporal fenestra with fossa; dermal bones of skull roof do not overhang rim at maturity (0) or dermal bones of skull roof overhang rim of supratemporal fenestra near maturity (1) or supratemporal fenestra closes during ontogeny (2).

(153) Shallow fossa at anteromedial corner of supratemporal fenestra (0) or no such fossa; anteromedial corner of supratemporal fenestra smooth (1).

(154) Medial parietal wall of supratemporal fenestra imperforate (0) or bearing foramina (1).

(155) Parietal and squamosal widely separated by quadrate on posterior wall of supratemporal fenestra (0) or parietal and squamosal approach each other on posterior

wall of supratemporal fenestra without actually making contact (1) or parietal and squamosal meet along posterior wall of supratemporal fenestra (2).

(156) Skull table surface slopes ventrally from sagittal axis (0) or planar (1) at maturity.

(157) Posterolateral margin of squamosal horizontal or nearly so (0) or upturned to form a discrete horn (1).

(158) Mature skull table with broad curvature; short posterolateral squamosal rami along paroccipital process (0) or with nearly horizontal sides; significant posterolateral squamosal rami along paroccipital process (1).

(159) Squamosal does not extend (0) or extends (1) ventrolaterally to lateral extent of paraoccipital process.

(160) Supraoccipital exposure on dorsal skull table small (0), absent (1), large (2), or large such that parietal is excluded from posterior edge of table (3).

(161) Anterior foramen for palatine ramus of cranial nerve VII ventrolateral (0) or ventral (1) to basisphenoid rostrum.

(162) Sulcus on anterior braincase wall lateral to basisphenoid rostrum (0) or braincase wall lateral to basisphenoid rostrum smooth; no sulcus (1).

(163) Basisphenoid not exposed extensively (0) or exposed extensively (1) on braincase wall anterior to trigeminal foramen.

(164) Extensive exposure of prootic on external braincase wall (0) or prootic largely obscured by quadrate and laterosphenoid externally (1).

(165) Laterosphenoid bridge comprised entirely of laterosphenoid (0) or with ascending process or palatine (1).

(166) Capitate process of laterosphenoid oriented laterally (0) or anteroposteriorly (1) toward midline.

(167) Parietal with recess communicating with pneumatic system (0) or solid, without recess (1).

(168) Significant ventral quadrate process on lateral braincase wall (0) or quadrate-pterygoid suture linear from basisphenoid exposure to trigeminal foramen (1).

(169) Lateral carotid foramen opens lateral (0) or dorsal (1) to basisphenoid at maturity.

(170) External surface of basioccipital ventral to occipital condyle oriented posteroventrally (0) or posteriorly (1) at maturity.

(171) Posterior pterygoid processes tall and prominent (0) or small and project posteroventrally (1) or small and project posteriorly (2).

(172) Basisphenoid thin (0) or anteroposteriorly wide (1) ventral to basioccipital.

(173) Basisphenoid not broadly exposed ventral to basioccipital at maturity; pterygoid short ventral to median eustachian opening (0) or basisphenoid exposed as broad sheet ventral to basioccipital at maturity; pterygoid tall ventral to median eustachian opening (1).

(174) Exoccipital with very prominent boss on paroccipital process; process lateral to cranioquadrate opening short (0) or exoccipital with small or no boss on paroccipital process; process lateral to cranioquadrate opening long (1).

(175) Lateral eustachian canals open dorsal (0) or lateral (1) to medial eustachian canal.

(176) Exoccipitals terminate dorsal to basioccipital tubera (0) or send robust process ventrally and participate in basioccipital tubera (1) or send slender process ventrally to basioccipital tubera (2).

(177) Quadrate foramen aerum on mediodorsal angle (0) or on dorsal surface (1) of quadrate.

(178) Quadrate foramen aereum is small (0), comparatively large (1), or absent (2) at maturity.

(179) Quadrate lacks (0) or bears (1) prominent, mediolaterally thin crest on dorsal surface of ramus.

(180) Attachment scar for posterior mandibular adductor muscle on ventral surface of quadrate ramus forms modest crests (0) or prominent knob (1).

(181) Quadrate with small, ventrally-reflected medial hemicondyle (0) or with small medial hemicondyle; dorsal notch for foramen aerum (1) or with prominent dorsal projection between hemicondyles (2) or with expanded medial hemicondyle (3).

(182) Iris (0) greenish/yellowish or (1) brown.

(183) Two or more (0) or one (1) row of postoccipital osteoderms.

(184) Fewer than eight (0) or eight to 14 (1) or more than 14 (2) paired midline scale rows (there is considerable variation in this character within species, and further work is needed to clarify the situation; data from Fuchs, 2006.)

(185) Ectopterygoid maxillary ramus forms less than (0) or more than (1) two-thirds of lateral margin of suborbital fenestra.

(186) Ectopterygoid maxillary ramus terminates at lateral margin of suborbital fenestra (0) or lateral to it, with maxilla separating the ectopterygoid from fenestra for short distance.

(187) Palatine-maxillary suture intersects suborbital fenestra at its anteromedial margin (0) or nearly at its anteriormost limit (1).

(188) Frontal lacks (0) or bears (1) prominent midsagittal crest between orbits.

(189) All cervical neural spines anteroposteriorly broad (0) or posterior neural spines thin and rod-like (1).
