# Supplementary material for: New Crocodyliforms from Southwestern Europe and Definition of a Diverse Clade of European Late Cretaceous Basal Eusuchians
Source: PLoS One. 2015 Nov 4;10(11):e0140679. doi: 10.1371/journal.pone.0140679 (PMC4633049; doi:10.1371/journal.pone.0140679)
Supplement: S3 Supporting Information — (PDF) [file pone.0140679.s003.pdf]

S3. List of several measurements of the skull of the holotypes of the two known species of *Lohuecosuchus*, HUE-04498 (*L. megadontos*) and MDE/CM-616 (*L. mechinorum*)

**Measurements of the skull (mm)**	**HUE-04498**	**MDE/CM-616**
Skull, maximum length from anterior end of premaxillae to quadrate condyles	290	385
Skull, medial length from anterior end of premaxillae to posterior end of supraoccipital	245	310
Skull, maximum width between lateral margins of the quadratojugals	182	285
Snout, maximum width at the level of the anterior end of the orbits	98	220
Snout, medial length from anterior end of premaxillae to the anterior end of the orbits	111	182
Supratemporal fenestra, anteroposterior length	32	30
Supratemporal fenestra, lateromedial width	23	46
Infratemporal fenestra, ventral border	40	48
Naris, length	18	34
Naris, width	32	56
Choana, length	16	55
Choana, width	16	20
Orbit, length	41	54
Orbit, width	43	58
Suborbital fenestra, anteroposterior length	76	120
Suborbital fenestra, maximum width	28	47
